# On the Microstructure and Properties of Nb-Ti-Cr-Al-B-Si-X (X = Hf, Sn, Ta) Refractory Complex Concentrated Alloys

**DOI:** 10.3390/ma14247615

**Published:** 2021-12-10

**Authors:** Tophan Thandorn, Panos Tsakiropoulos

**Affiliations:** 1 Department of Materials Science and Engineering, School of Science, Mae Fah Luang University, Chiang Rai 57100, Thailand; tophan@mfu.ac.th; 2 Department of Materials Science and Engineering, Sir Robert Hadfield Building, The University of Sheffield, Sheffield S1 3JD, UK

**Keywords:** high-entropy alloys, complex concentrated alloys, refractory metal intermetallic composites, Nb silicide-based alloys, alloy design, oxidation

## Abstract

We studied the effect of the addition of Hf, Sn, or Ta on the density, macrosegregation, microstructure, hardness and oxidation of three refractory metal intermetallic composites based on Nb (RM(Nb)ICs) that were also complex concentrated alloys (i.e., RM(Nb)ICs/RCCAs), namely, the alloys TT5, TT6, and TT7, which had the nominal compositions (at.%) Nb-24Ti-18Si-5Al-5B-5Cr-6Ta, Nb-24Ti-18Si-4Al-6B-5Cr-4Sn and Nb-24Ti-17Si-5Al-6B-5Cr-5Hf, respectively. The alloys were compared with B containing and B free RM(Nb)ICs. The macrosegregation of B, Ti, and Si was reduced with the addition, respectively of Hf, Sn or Ta, Sn or Ta, and Hf or Sn. All three alloys had densities less than 7 g/cm^3^. The alloy TT6 had the highest specific strength in the as cast and heat-treated conditions, which was also higher than that of RCCAs and refractory metal high entropy alloys (RHEAs). The bcc solid solution Nb_ss_ and the tetragonal T2 and hexagonal D8_8_ silicides were stable in the alloys TT5 and TT7, whereas in TT6 the stable phases were the A15-Nb_3_Sn and the T2 and D8_8_ silicides. All three alloys did not pest at 800 °C, where only the scale that was formed on TT5 spalled off. At 1200 °C, the scale of TT5 spalled off, but not the scales of TT6 and TT7. Compared with the B free alloys, the synergy of B with Ta was the least effective regarding oxidation at 800 and 1200 °C. Macrosegregation of solutes, the chemical composition of phases, the hardness of the Nb_ss_ and the alloys, and the oxidation of the alloys at 800 and 1200 °C were considered from the perspective of the Niobium Intermetallic Composite Elaboration (NICE) alloy design methodology. Relationships between properties and the parameters VEC, δ, and Δχ of alloy or phase and between parameters were discussed. The trends of parameters and the location of alloys and phases in parameter maps were in agreement with NICE.

## 1. Introduction

New materials are needed to replace Ni based superalloys in the hottest parts of aero engines to enable them to meet stringent environmental and performance targets in the future. These ultra-high temperature materials (UHTMs) can be metallic materials, such as refractory metal (RM), intermetallic composites (RMICs), RM high entropy alloys (RHEAs), or RM complex concentrated alloys (RCCAs). The UHTMs must meet specific property targets about toughness, creep and oxidation [1].

To date, different alloying elements have been reported in RM(Nb)ICs, i.e., the RMICs based on Nb, and in RHEAs or RCCAs, albeit not all in the same metallic UHTM. In RM(Nb)ICs these alloying additions are simple metal (SM) and metalloid (Met) elements, rare-earth elements, transition metals (TMs), and RMs and, to our knowledge, include Al, B, *C, Ce*, Cr, *Dy, Er, Fe, Ga*, Ge, Hf, *Ho, In*, Mo, Nb, Si, Sn, Ta, Ti, V, W, Y, and Zr, where the elements that are not currently used in RHEAs or RCCAs are shown in italics [1,2]. The RCCAs use the transition and refractory metals Cr, Hf, Mo, Nb, Re, Ta, V, W, and Zr and can also contain Al, Co, Ni, Si, Ti, C, or N [2]. The latter seven elements may form intermetallic phases that may increase the strength and hardness, may improve oxidation, and can decrease the density of RCCAs [2]. The addition of B, Fe, or Y in RCCAs also has been suggested [2]. In other words, RHEAs, RCCAs, and RM(Nb)ICs essentially use the same alloying elements. However, it should be noted that the elements B, Ge, or Sn, which are not shown in italics above, currently are used only in RM(Nb)ICs and in RM(Nb)ICs that are also RCCAs (i.e., RM(Nb)ICs/RCCAs) [1,3,4]. The addition of B, Ge, or Sn, together with Al, Cr, Hf, Si, or Ti can significantly improve oxidation resistance in the pest oxidation regime and at high temperatures [4,5,6], and thus these elements can assist the alloy designer to develop new metallic UHTMs that offer a balance of properties [1,3,4,5,6,7,8,9].

The B addition in metallic UHTMs requires special attention. To start with, in the Nb-Si-B system [10] B forms two 5-3 silicides with Nb and Si, namely, (a) the tetragonal T2, which has the same crystal structure as the αNb_5_Si_3_ (tI32, Cr_5_B_3_-type, D8_l_); and (b) the hexagonal D8_8_ silicide, which has the same crystal structure as the γNb_5_Si_3_ (hP16, Mn_5_Si_3_-type, D8_8_), i.e., the metastable 5-3 silicide in the Nb-Si binary [11]. The two 5-3 silicides are referred to as T2 and D8_8_. Furthermore, in plots of diffusivity or Young’s elastic modulus versus electronegativity (χ) or atomic size (r), B belongs in the same group with some of the other elements given above [3,12]. Additionally, in the Δχ versus δ and Δχ versus VEC master maps of RM(Nb)ICs, RM(Nb)ICs/RCCAs, and RCCAs with Nb addition and their phases [1,3,4,13]: (i) the alloys with B addition occupy a distinct area separate from the areas of the B free metallic UHTMs; (ii) the B containing bcc Nb solid solutions are located in a distinct part of the area of the bcc Nb solid solutions in RM(Nb)ICs, RM(Nb)ICs/RCCAs, and bcc solid solution RCCAs with Nb addition; and (iii) the B containing tetragonal Nb_5_Si_3_ (i.e., the T2 silicide) also is found in a separate region of the area occupied by the tetragonal Nb_5_Si_3_ silicide (for the parameters δ (based on atomic size r), Δχ (based on electronegativity χ), and VEC (number of valence electrons per atom filled into the valence band) see below in this section). Moreover, in the Δχ versus VEC map of the tetragonal Nb_5_Si_3_ silicide [4,14], when the Si in the silicide is substituted by B, the silicide shifts in the opposite direction compared with the substitution of Si by Ge or Sn.

Let us now consider the following questions. How does one design/select a RCCA with a balance of properties and how does s/he choose which alloying elements to use? Should the RCCA be a solid solution(s) alloy or a multiphase alloy? In the latter case which should be the desirable phases? If the approach adopted in [15] were to be followed, the alloy designer can say very little, if anything, about phase(s) and properties in the design stage. The same would be the case if only the TMs and RMs of the groups 4, 5, and 6 were to be considered (i.e., the elements Ti, Zr, Hf, V, Nb, Ta, and Cr, Mo, and W). In the latter case solid solution RCCAs are likely [2] but, in some way, s/he will have to choose alloy(s) from among 382 quaternary and higher order alloy systems with up to nine elements. Oxidation resistance is unlikely with the TMs and RMs of groups 4, 5, and 6 [2,3]. Therefore, if s/he were to expand his/her choice of alloying elements, say, consider, the alloying elements Al, B, Ge, Si, and Sn with TM = Cr, Hf, Ti, and RM = Mo, Nb, Ta, and W, i.e., twelve elements, then, by some means, s/he will have to choose alloy(s) from among 3797 quaternary and higher order alloy systems with up to 12 elements. In his/her alloy design/selection s/he will be confronted with the lack of and conflicting reports about thermodynamic data [1,2,3].

The new alloy design methodology NICE (Niobium Intermetallic Composite Elaboration) [3] can assist the alloy designer in his/her task to design/select suitable metallic UHTMs for development. NICE was developed for RM(Nb)ICs and has been extended and applied to RM(Nb)ICs/RCCAs, and RHEAs and RCCAs with Nb addition [1,3,4]. NICE is the outcome of an overall conception for the design of RMICs, RHEAs, and RCCAs that is systematic and coherent. Its foundations were set up in five papers [12,13,14,16,17]. For alloys and solid solutions NICE uses relationships between actual alloy or phase composition and the parameters δ, Δχ, ΔH_mix_ (enthalpy of mixing), ΔS_mix_ (entropy of mixing), VEC, and Ω (= T_m_ΔS_mix_/|ΔH_mix_|) (the same parameters are used in the study of HEAs [2,12,13,18]). For intermetallics in the alloys, namely, M_5_Si_3_ silicides, C14-NbCr_2_ Laves, and A15-Nb_3_X (X = Al, Ge, Si, and Sn) compounds, NICE uses relationships between actual phase composition and the phase parameters δ, Δχ, and VEC [14,16,17]. NICE is a property driven alloy design methodology. Relationships between the aforementioned parameters of alloys, their solid solution(s) and intermetallic(s) and properties, such as yield strength, hardness, creep, or oxidation are used in NICE to design alloys to meet property targets [1,3,4,19,20,21,22,23].

The papers [1,3,4] described how NICE operates. They showed how a large volume of experimental data about actual compositions of alloys, their phases and properties can be used to deal with essential questions about the design of RMICs, RHEAs, and RCCAs. In other words, NICE allows us to address the whole problem of the development of metallic UHTMs, i.e., RM(Nb)ICs, RM(Nb)ICs/RCCAs, RHEAs, and RCCAs, as one materials system.

The significations of NICE cannot be understood in isolation from the development of metallic UHTMs, with which it is linked [1,3,4]. It is based on knowledge that constantly gives rise to new knowledge [1,4]. New data can guide a “fine-tuning” of this new alloy design method [1,3,4]. The very object of NICE is the new. NICE is about the factors (parameters) that have a practical importance in alloy development, e.g., it guides the selection of alloys that are worthy of further research and development, for example, see [20,21,22]. With NICE, precise answers to a host of questions are possible but not to all questions.

NICE is a “property goal driven” alloy design approach that leads to the selection of metallic UHTMs worthy of development owing to promising oxidation and/or creep properties [1,3,4]. It does not consider toughness. Moreover, it does not consider the “architecture” of the microstructure, meaning distribution of phases and their size and shape, which can be altered by processing that can improve properties [1]. In NICE the design of an alloy combines property target(s), say a creep rate (έ) target or a mass change (ΔW/A) target in oxidation, (e.g., [21,24,25]) with constraint(s), say, desirable alloying elements and/or phases or RM/TM, or SM/Met ratios in the alloy (e.g., see [19,20,21,24]).

The motivation for the research presented in this paper was to design/select and study B and Nb containing RCCAs in order to assist the development of metallic UHTMs with a balance of properties. This paper is about three B containing RM(Nb)ICs/RCCAs, each with seven elements. The alloy design methodology NICE was used with (i) two property targets, namely, (a) minimum room temperature yield strength (σ_y_^RT^) of 2000 MPa; and (b) mass change (ΔW/A) of 5 and 25 mg/cm^2^ in isothermal oxidation, respectively at 800 and 1200 °C, and (ii) four constraints, namely, (a) Ti/Si = 1.4; (b) desirable stable phases bcc Nb_ss_ and the tetragonal T2 and hexagonal D8_8_ silicides; (c) the alloys to be in the area C in the Δχ versus δ master map of metallic UHTMs [1]; and (d) the macrosegregation of Si and Ti to be less than 5 and 4 at.%, respectively.

The selection of the property targets was “advised” from the results in [21,24] and [26,27] and of the constraints (iia) from [1,27], (iib) from [26,27], (iic) from [1,4,13] and (iid) from [27]. From the possible 792 alloy systems with 7 elements, NICE gave us a number of metallic UHTMs, of which we report in this paper the results of three RM(Nb)ICs/RCCAs with the addition of Hf, Sn, or Ta, which were selected for two reasons, namely, (1) to allow comparison of the alloy microstructures and properties with the “reference” B containing RM(Nb)IC alloy TT4 [27] and three “reference” B free RM(Nb)ICs, namely, KZ6 [28] (compared with TT5), ZX8 [29] (compared with TT6), and JN1 [30] (compared with TT7); and (2) because Hf and Sn are two key elements that improve oxidation when in synergy with Al, Cr, and Ti [1,3,4]. For the nominal compositions of the reference alloys see the Appendix A. The three alloys of this study are the alloys TT5, TT6, and TT7 with nominal compositions (rounded numbers, at.%), respectively 37Nb-24Ti-18Si-5Al-5B-5Cr-6Ta (36.9Nb-23.8Ti-18.1Si-5.2Al-5B-5.1Cr-5.9Ta), 39Nb-24Ti-18Si-4Al-6B-5Cr-4Sn (39.1Nb-24Ti-17.8Si-4Al-6.1B-5.1Cr-3.9Sn), and 38Nb-24Ti-17Si-5Al-6B-5Cr-5Hf (38.4Nb-23.9Ti-17.2Si-4.8Al-6B-4.9Cr-4.8Hf), where in the parentheses are given the exact calculated compositions.

The structure of the paper is as follows. The description of experimental procedures is followed by the presentation of the results for the microstructures, hardness of the alloys, and preliminary results of their oxidation at 800 and 1200 °C. The discussion of the results includes their scrutiny from the perspective of NICE.

## 2. Experimental

The alloys (Table 1) were produced as large button ingots (0.6 kg) in an argon atmosphere from high purity (≥99.9 wt.%) Al, B, Cr, Hf, Nb, Si, Sn, Ta, and Ti using arc melting with a non-consumable tungsten electrode and a water-cooled copper crucible. Each alloy was melted five times. The alloys were heat treated for 100 h at 1500 °C. The specimens were wrapped in Ta foil and were heat treated in a tube furnace under a continuous flow of Ti gettered argon followed by furnace cooling to room temperature. The characterisation of the microstructures was conducted using an XRD (Hiltonbrooks Ltd., Crewe, UK, Philips diffractometer, Cu radiation, solid specimens, JCPDS database) and microanalysis using EPMA (JEOL 8600 electron probe micro-analyser, JEOL, Tokyo, Japan, operating conditions 9 kV with a beam current of 30 nA [31]) equipped with WDX and EDX spectrometers, elemental standards and BN and TiN standards, in the same way with [26,27]. Standard metallographic procedures were used to prepare specimens. These were mounted in Bakelite and ground using SiC grinding papers (320 to 2400 grit) followed with polishing using diamond DUR clothes (6 and 1 μm finish). The part of the button ingot in contact with the water-cooled copper crucible is referred to as bottom of the ingot in this paper.

The density of the alloys was measured using the Archimedes principle and a Sartorious electronic precision balance (Sartorius Lab Instruments GmbH & Co. KG, Göttingen, Germany), equipped with a density measurement kit. The isothermal oxidation of the as cast (AC) alloys was studied for up to 100 h at 800 and 1200 °C using Stanton Redcroft thermo-balances (Thermal Scientific plc., Odessa, TX, USA) and about 3 × 3 × 3 mm^3^ specimens from the as cast alloys. The weight of each sample was measured at the start and end of each oxidation experiment. A Mitutoyo HM-101 hardness testing machine (Mitutoyo America, Aurora, IL, USA) with Vickers indenter was used to measure the hardness (0.5 kg load) of the alloys and the microhardness (0.025 kg load) of the solid solution and T2 silicide. At least ten measurements were taken for each alloy and condition, and each phase. The area fraction of the solid solution was measured using the software KS Run 3 in a Polyvar Met microscope (Leica, Reichert Division, Wien, Austria). The SEM images of the large areas (X250) of the AC and heat treated (HT) alloys that were analysed by EPMA were used for the measurements.

The aforementioned parameters of the solid solution, 5-3 silicide and the alloys were calculated as described respectively in the refs [12,13,14]. Analysis of macrosegregation data was conducted as discussed in [32].

## 3. Results

The actual composition of each alloy in the AC or HT condition is given in Table 1. The densities of the alloys are given in Table 2. The alloy TT6 had the lowest density and the densities of all three alloys were lower than 7 g/cm^3^. The macrosegregation of solutes (MACX, X = Al, B, Cr, Si, Sn, and Ti) is given in Table 3. The highest MACSi and MACTi were observed, respectively in the alloys TT5 and TT6, whereas MACB did not differ significantly in these three alloys. The phases that were observed in the alloys using XRD and EPMA are summarised in Table 4, which also includes data for the “reference” alloys KZ6, ZX8, and JN1 (see introduction). The chemical compositions of the phases that were observed in all parts of the ingots are given in Table 5. In the latter are given the average value, the standard deviation and the minimum and maximum analysis values.

### 3.1. As Cast Alloys

**Figure 1:**
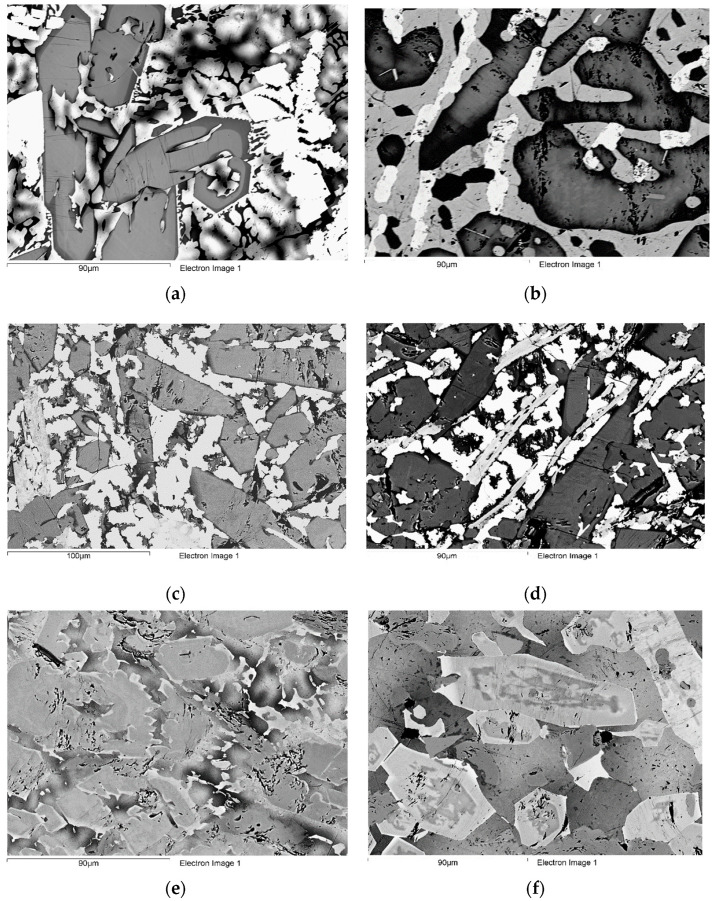
Microstructures of the AC (left hand side images) and HT (right hand side images) alloys TT5 (**a**,**b**), TT6 (**c**,**d**), and TT7 (**e**,**f**). Note that contrast enhancement has been applied to show different phases. See text for description of microstructures.

**Alloy TT5**: The microstructure of TT5-AC consisted of five phases, namely, Nb_ss_; the silicides T2, D8_8_, and Nb_3_Si; and a Cr and Ti rich phase; and also, a small vol.% of Nb_ss_ + T2 eutectic, see Table 4; Figure 1a and Figure S1a in the Supplemental Data. The chemical compositions of the phases are given in Table 5. Note that the D8_8_ silicide was Al free. The Nb_3_Si silicide was observed only in the top and bottom parts of the ingot and its average composition was 24.9Nb-38.1Ti-21.2Si-1.2B-8.0Cr-4.3Al-2.4Ta (at.%). The Cr and Ti rich phase was observed only in the top and bulk parts of the ingot and its average composition was 19.1Nb-37.2Ti-10.1Si-27.8Cr-3.4Al-2.3Ta (at.%). Note that this phase was B free. The Ta concentration in T2 decreased with increasing Ti content.

In Figure 1a, the grey facetted phase is the T2 silicide, the Ti rich T2 is the darker grey areas that are surrounded by a bright phase (Nb_ss_) with darker contrast areas (Ti rich Nb_ss_). The very bright phase is the D8_8_ silicide. There were no Ti rich areas in the D8_8_ phase. In the Nb_ss_ the concentrations of Al and Cr increased with Ti concentration (this was particularly strong for Cr) but the opposite was the case for B and Ta. The concentration of B in the Nb_ss_ varied significantly and there were B free Nb_ss_ and Ti rich Nb_ss_ grains.

The T2 silicide was facetted in all part of the ingot and severely cracked with the cracks running parallel to each other inside each grain but not extending in the Nb_ss_ and D8_8_ silicide. The average Si + B + Al concentrations in the T2 and Ti rich T2 were 37.8 and 37 at.%, and the Si + B content of the D8_8_ was 40 at.% (Table 6). The T2 and Ti rich T2 were poorer in B compared with the D8_8_ silicide (Table 5). Moreover, the Si concentration in the T2 and Ti rich T2 was higher than the B concentration, thus the Si/B ratio was 4.7, 5.1, and 0.55, respectively for the T2, Ti rich T2, and D8_8_ (Table 6).

**Alloy TT6**: The microstructure of TT6-AC consisted of five phases, namely, Nb_3_Sn, Nb_ss_, and the T2, D8_8_, and Nb_3_Si silicides, with a very small vol.% of Nb_ss_ + T2 eutectic (Table 4, Figure 1c and Figure S1c in the Supplemental Data). The Nb_3_Si was found only in the bulk and its average composition was 27.8Nb-40.4Ti-19.4Si-1.4B-5Cr-4.1Al-1.9Sn (at.%). The compositions of the other phases are given in Table 5. Note that the D8_8_ was Al and Sn free. The Nb_ss_ was Ti rich and B free and its vol.% was lower than that of the Nb_3_Sn compound (note that only the vol.% of Nb_3_Sn is given in Table 2). The vol.% of the D8_8_ silicide was significantly lower compared with TT5-AC. In Figure 1c, the grey phase is the T2 silicide, in which the dark contrast areas correspond to Ti rich T2. The Nb_3_Sn is the very bright contrast phase and the D8_8_ is the slightly darker contrast phase (in the middle of the bottom and in the middle of the left-hand side of Figure 1c) compared with the Nb_3_Sn.

The concentrations of Cr and Al increased with increasing Ti concentration in T2. The Sn solubility in the T2 was very low. The T2 exhibited strong faceting in all part of the ingot and was cracked but not as severely as in TT5-AC. The average Si + B + Al in T2 and Ti rich T2, respectively was 38.6 and 37.5 at.% and the average Si + B + Al + Sn was 39.2 and 38.3 at.%, respectively, whereas in the D8_8_ the Si + B content was 42.4 at.% (Table 6). The Si/B ratio in the T2, Ti rich T2, and D8_8_ silicides was 4.3, 4.7, and 0.5, respectively (Table 6).

**Alloy TT7**: The microstructure of TT7-AC consisted of four phases, namely, the Nb_ss_, and the T2, D8_8_, and Nb_3_Si silicides. The vol.% of Nb_ss_ was significantly lower than in the alloys TT5 and TT6 (Table 2). There was no Nb_ss_ + T2 eutectic in all parts of the ingot (Table 4; Figure 1e and Figure S1e in the Supplementary Data). The Nb_3_Si was observed only in the top and bulk of the ingot and its average composition was 45Nb-27.7Ti-12.4Si-1.8B-5.3Cr-3.4Al-4.4Hf (at.%). The compositions of the phases are given in Table 5. Compared with the alloys TT5 and TT6 in which the D8_8_ silicide was also observed, the segregation behaviour of Hf (see below) in TT7-AC made difficult both the imaging and the analysis of the D8_8_ silicide using EPMA. Furthermore, the vol.% of the D8_8_ silicide was low in TT7-AC and lower than in TT6-AC. The D8_8_ silicide was observed in all parts of the ingot, but analyses were possible only in the bulk (average composition 46Nb-10.6Ti-13.3Si-26.9B-1.2Cr-2Hf (at.%)) and top (average composition 45Nb-12Ti-12.7Si-27.7B-0.8Cr-1.8Hf (at.%)) of the ingot. Note that the D8_8_ was also Al free in TT7-AC. There were no Ti rich areas in the D8_8_ silicide.

In Figure 1e, the large grey contrast grains are the T2 silicide, with the darker grey contrast areas the Ti rich T2. The T2 grains are surrounded by the bright contrast D8_8_ silicide and the Nb_ss_ with grey contrast (similar to that of the D8_8_ silicide) and Ti rich Nb_ss_ with darker grey contrast. The concentrations of Al and Cr in the Nb_ss_ increased with Ti concentration but the opposite was the case for B. The concentration of Hf in the Nb_ss_ did not change with its Ti content. The concentration of B in the Nb_ss_ varied significantly and some Nb_ss_ grains and Ti rich Nb_ss_ were B free. The Hf concentration in the T2 decreased with the increase in Ti content. The faceting of theT2 was not very strong compared with the alloys TT5 and TT6. Furthermore, the T2 silicide was not cracked. There was strong variation of the B content in the silicides. In the Ti rich T2 the B concentration decreased, in agreement with the results for the alloys TT5 and TT6. The average Si + B + Al concentration in the T2 and Ti-rich T2 was 39.3 and 37.5 at.%, respectively (Table 6). In the D8_8_ the average Si + B content was about 40.3 at.% (Table 6). The Si/B ratio in T2, Ti rich T2 and D8_8_ was 5, 6.1, and about 0.475, respectively (Table 6).

### 3.2. Heat Treated Alloys

**Alloy TT5**: There was significant homogenisation of TT5-HT, its composition was closer to the nominal one (Table 1) and its microstructure was not contaminated by nitrogen (i.e., no TiN was observed). The Nb_ss_, and the T2, Ti rich T2, and D8_8_ silicides were observed (Table 5; Figure 1b and Figure S1b in the Supplemental Data). The vol.% of Nb_ss_ increased compared with TT5-AC (Table 2). In Figure 1b, the dark grey phase is the T2 silicide, the grey phase is the Nb_ss_, and the bright contrast phase is the D8_8_ silicide. Both the T2 and D8_8_ were not facetted and not cracked. The D8_8_ silicide was Al free. Ti rich T2 was still present after the heat treatment (Table 5). The concentration of Ti in the Ti rich T2 had decreased from 32.8 to 25.7 at. %, whereas in the D8_8_ the Ti concentration essentially was not changed (Table 5). The Si + B + Al content in T2 and Ti rich T2, respectively was 37 and 38 at.%, but the D8_8_ had Si + B = 40 at.%. The Si/B ratio in T2, Ti rich T2 and the D8_8_ silicide, respectively was 4.2, 4.8, and 0.53 (Table 6).

**Alloy TT6**: The chemical composition of TT6-HT was close to the nominal one (Table 1) and the alloy was not contaminated by nitrogen. The microstructure consisted of Nb_3_Sn, and the T2 and D8_8_ silicides (Table 5; Figure 1d and Figure S1d in the Supplemental Data). The latter two silicides exhibited no strong faceting and were not cracked. There was no Nb_ss_ and the vol.% of Nb_3_Sn had increased significantly (Table 2). The D8_8_ was Al free and the chemical compositions of Nb_3_Sn and D8_8_ essentially were the same with those in TT6-AC. In the Ti rich T2 the average B content essentially did not change but the standard deviation increased (Table 5). In the T2 and Ti rich T2 the Si + B + Al content respectively was 36.6 and 37.1 at.%, the Si + B + Al + Sn content, respectively was 37.2 and 38.1 at.%, and the D8_8_ silicide had Si + B = 40.1 at.%. The Si/B ratio in T2, Ti rich T2, and D8_8_, respectively was 3.9, 5.4, and 0.43 (Table 6).

**Alloy TT7**: There was significant homogenisation of the microstructure of TT7-HT, the chemical composition of which was close to the nominal one (Table 1) and no contamination by nitrogen. The microstructure consisted of the Nb_ss_, and the T2, Ti rich T2, and D8_8_ silicides (Table 5; Figure 1f and Figure S1f in the Supplemental Data). The vol.% of the D8_8_ was very low but the vol.% of Nb_ss_ had significantly increased (Table 2). Some grains of T2 contained cracks. Solute partitioning between the phases made their identification under back scatter electron (BSE) imaging conditions very difficult. In Figure 1f, the large grey contrast grains with darker contrast areas in their bulk are the T2 silicide. With reference the large T2 grain observed slightly above the middle of the image, the bright contrast phase is the D8_8_ silicide, the darker grey contrast grain above the D8_8_ is Ti rich T2 and the slightly lighter grey contrast grain to the left of the D8_8_ and Ti rich T2 is the Nb_ss_. The Si + B + Al content in the T2 and Ti rich T2 respectively was 37.5 and 36.6 at.% and the Si + B in D8_8_ was 40 at.%. The Si/B ratio in T2, Ti rich T2, and D8_8_ was 4.3, 7 and 0.47, respectively (Table 6).

### 3.3. Hardness

The hardness of the alloys and the microhardness of phases are given in Table 2, where the data gives the average value, standard deviation, and minimum and maximum hardness values. The alloys TT6 and TT7 had the highest hardness in the as cast condition (776 HV), and the alloy TT6 exhibited the smaller reduction in hardness after the heat treatment. The Nb_ss_ and T2 silicide had the highest microhardness in the as cast condition and the T2 in TT5 retained its microhardness after the heat treatment, and its hardness was higher than the hardness of Nb_5_Si_3_ (1360 HV [14]). The microhardness of Nb_3_Sn in TT6 was significantly higher than that of the Nb_ss_ in TT5 and TT6 (Table 2).

### 3.4. Oxidation

**Figure 2:**
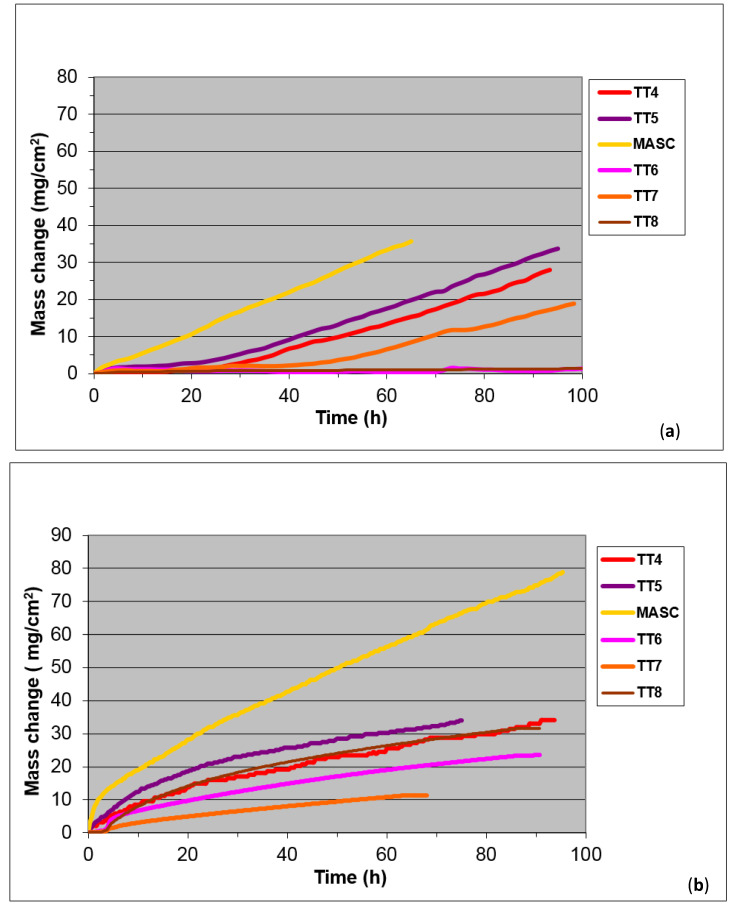
Mass change (ΔW/A) versus time of the alloys TT4 and TT8 [27], TT5, TT6, TT7, and MASC [27] at (**a**) 800 °C and (**b**) 1200 °C. Colours: red TT4, purple TT5, pink TT6, orange TT7, brown TT8, yellow MASC.

**Figure 3:**
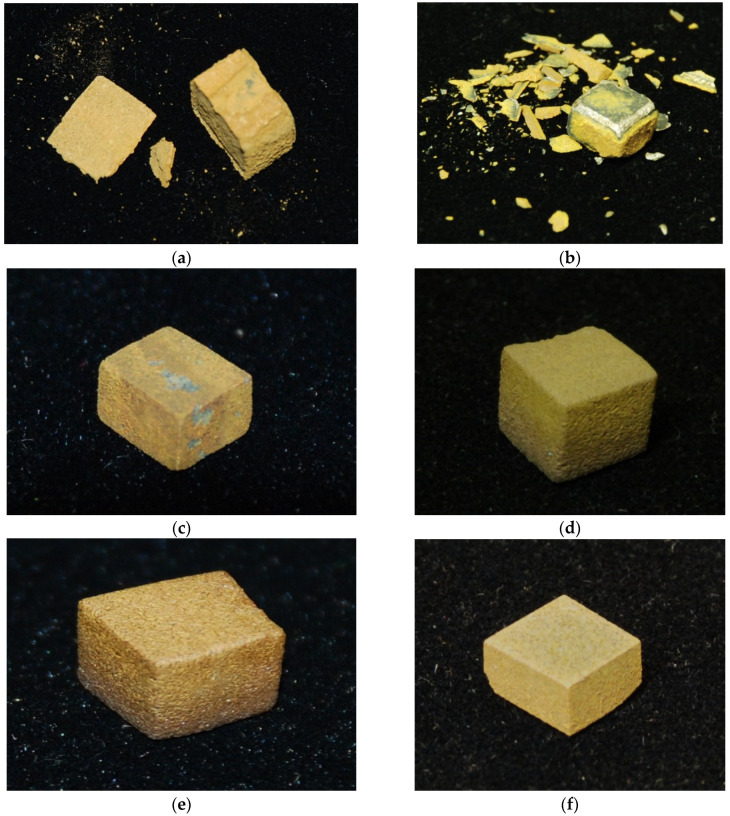
Specimens of the alloys TT5, TT6, and TT7 after isothermal oxidation (**a**,**c**,**e**) at 800 °C and (**b**,**d**,**f**) at 1200 °C. (**a**,**b**) alloy TT5, (**c**,**d**) TT6, (**e**,**f**) TT7. For dimensions of specimens see the experimental section.

The mass change for isothermal oxidation at 800 and 1200 °C for up to 100 h and images of the oxidised specimens are shown respectively in Figure 2 and Figure 3. For comparison purposes, Figure 2 includes data for other three RM(Nb)ICs, namely, the B free MASC alloy (Nb-25Ti-16Si-2Al-2Cr-8Hf [33]) and the alloys TT4 and TT8 [27]. Data for MASC are included because this is a “first generation” B free RM(Nb)IC and is often used as a “reference” alloy for comparison purposes. It should be noted that the microstructures of TT4 and TT8 consisted of the Nb_ss_ and the T2 and D8_8_ silicides [27]. The mass changes and oxidation rate constants are given in Table 7, which also gives data for the alloys TT4, TT8, and MASC. The alloys TT6 and TT8 gained the lowest weight at 800 °C whereas the alloys TT6 and TT7 gained the lowest weight at 1200 °C. None of the alloys with B addition suffered from pest oxidation at 800 °C. However, at 800 °C the scales that formed on the alloys TT4 and TT5 spalled off. At 1200 °C, scale spallation was also observed for the alloys TT4, TT5, and MASC. At 800 and 1200 °C, respectively each alloy followed linear and parabolic oxidation kinetics. The alloys TT6 and TT8 had the lowest k_l_ and the alloy TT7 had the lowest k_p_ value.

## 4. Discussion

### 4.1. Macrosegregation of B, Si and Ti

The synergy of B with Ta, Sn, or Hf in the alloys TT5, TT6, and TT7 affected its macrosegregation (MACB) and that of Si (MACSi) and Ti (MACTi). In Table 8, the macrosegregation of these three elements in the RM(Nb)ICs/RCCAs of this work, and the RM(Nb)ICs TT4 and TT8 [27], and KZ5 [34], KZ6 [28], JG3 [35], JN1 [30], and ZX8 [29] is compared. To understand the macrosegregation of solutes in B containing alloys, we use the RM(Nb)IC KZ5 as the reference alloy (see Appendix A for nominal alloy compositions, also note that JN1 = KZ5 + Hf, JG3 = KZ5 + Mo, KZ6 = KZ5 + Ta, ZX8 = KZ5 + Sn, TT4 = KZ5 + B, TT5 = KZ6 + B = KZ5 + Ta + B, TT6 = ZX8 + B = KZ5 + Sn + B, TT7 = JN1 + B = KZ5 + Hf + B, TT8 = JG3 + B = KZ5 + Mo + B, in other words each alloy is based on KZ5 plus specific alloying addition(s)). The data in Table 8 show: (i) that MACB was reduced in all three alloys, compared with TT8, to an average value of about 2.6 at.%; (ii) that the synergy (a) of B with Sn significantly reduced MACSi (compare KZ5, ZX8, and TT6), (b) of B with Ta slightly increased MACSi (compare KZ5, KZ6, and TT5), and (c) of B with Hf significantly reduced MACSi (compare KZ5, JN1, and TT7); and (iii) that the synergy (a) of B with Ta or Sn significantly reduced MACTi (compare KZ5, KZ6, TT5 and KZ5, ZX8, and TT6) and (b) of B with Hf slightly increased MACTi (compare KZ5, JN1, and TT7).

**Figure 4:**
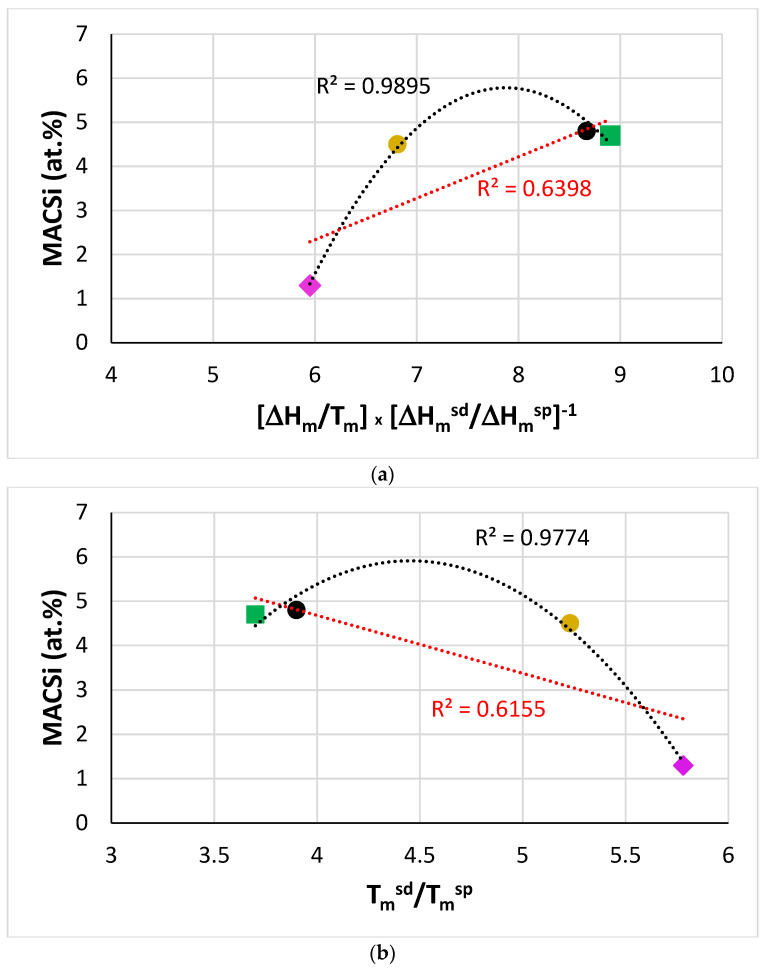
MACSi versus the parameters (**a**) [ΔH_m_/T_m_] × [ΔH_m_^sd^/ΔH_m_^sp^]^−1^ and (**b**) T_m_^sd^/T_m_^sp^. Alloys KZ5 (diamond), KZ6 (gold), TT4 (square), and TT5 (black). The parameters were calculated as described in [32].

Specific parameters [32] can describe macrosegregation of solutes in B free and B containing RM(Nb)ICs and RM(Nb)ICs/RCCAs [23,24,27,29,36]. For example, Figure 4 shows that when B was in synergy with Ta (alloy TT5), MACSi correlated with the parameters [ΔH_m_/T_m_] × [ΔH_m_^sd^/ΔH_m_^sp^]^−1^ and T_m_^sd^/T_m_^sp^ and that the trends were in agreement with [32]. For RM(Nb)ICs where B was in synergy with other solute additions, correlations of MACSi with these and other parameters can be found in [27]. Further research is needed to understand how the synergy of B with Sn or Hf affects MACSi.

**Figure 5:**
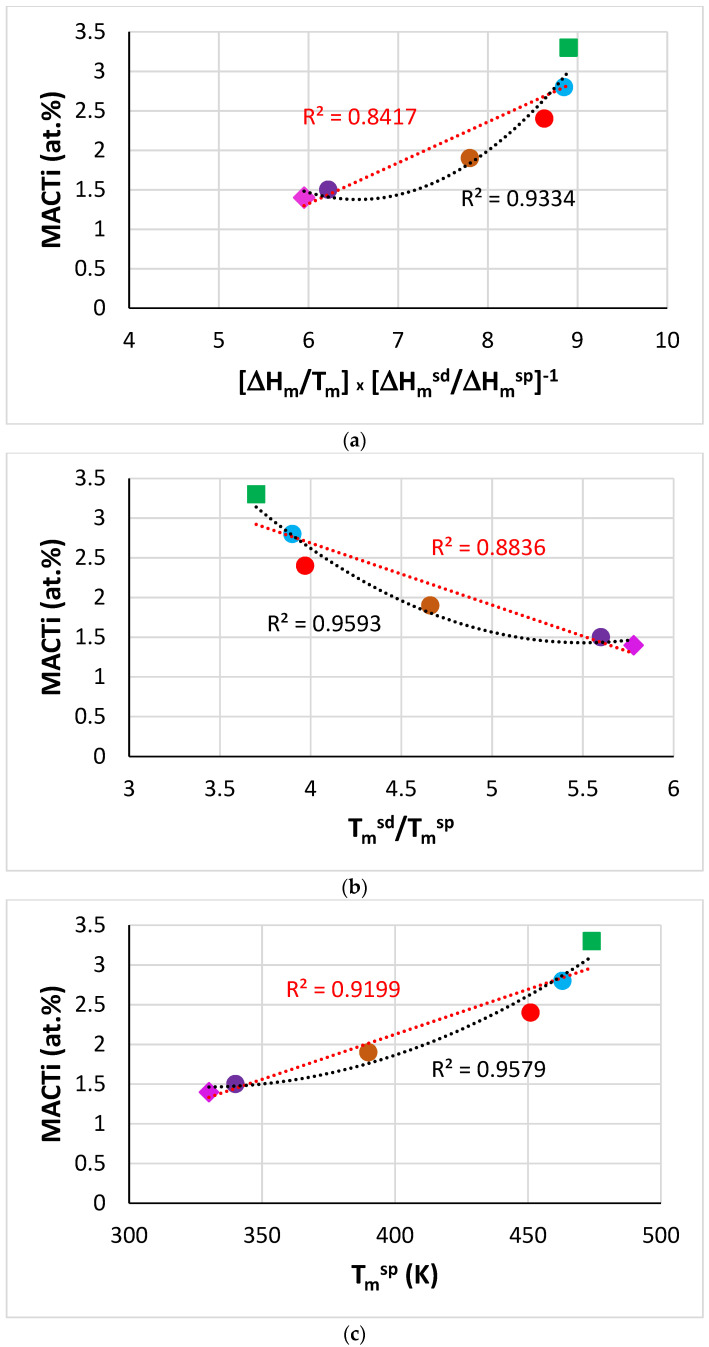

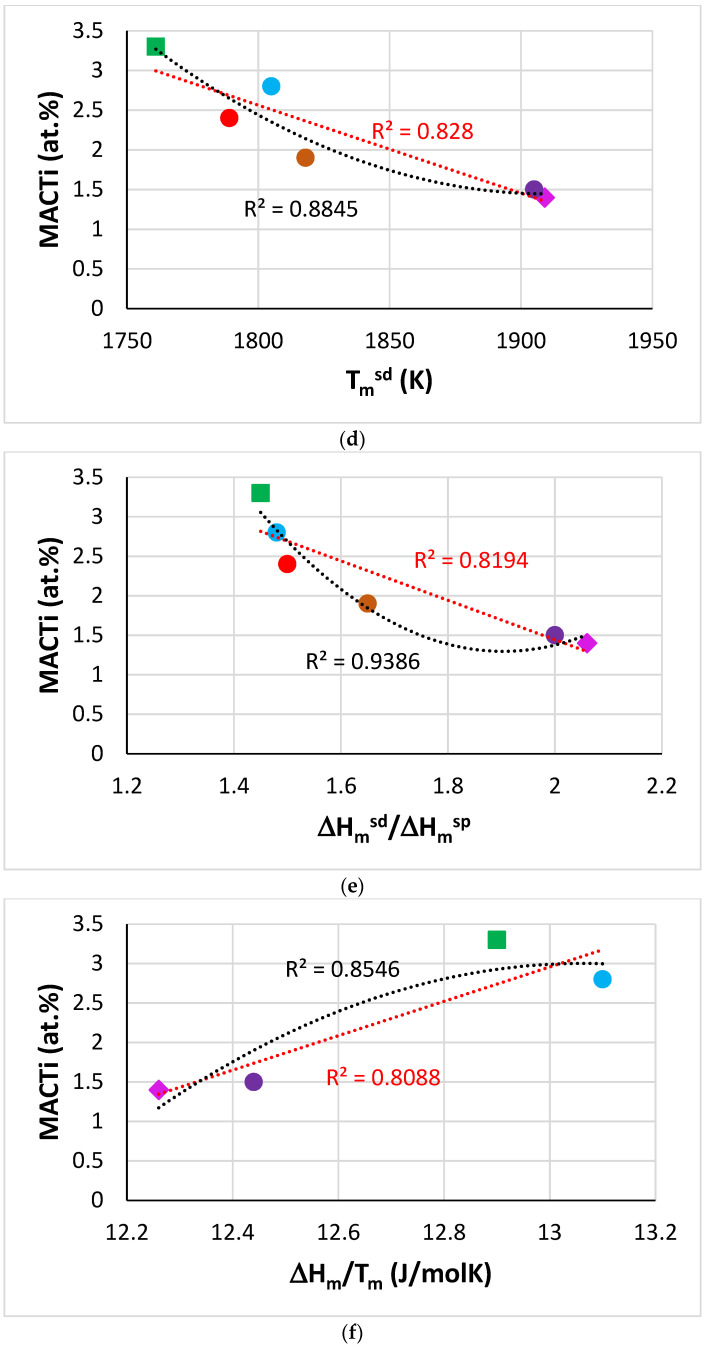
MACTi versus the parameters (**a**) [ΔH_m_/T_m_] × [ΔH_m_^sd^/ΔH_m_^sp^]^−1^, (**b**) T_m_^sd^/T_m_^sp^, (**c**) T_m_^sp^, (**d**) T_m_^sd^, (**e**) ΔH_m_^sd^/ΔH_m_^sp^ and (**f**) ΔH_m_/T_m_. The parameters were calculated as described in [32]. Alloys KZ5 (diamond), JN1 (brown), JG3 (purple), TT4 (square), TT7 (red), and TT8 (blue). In (**a**) R^2^ = 0.9334 is for the parabolic fit of all the data, for the alloys KZ5, JN1, TT4 and TT7 the R^2^ values were R^2^ = 0.7761 (linear fit) and R^2^ = 0.9267 (parabolic), whereas for the alloys KZ5, JG3, TT4 and TT8, R^2^ = 0.9582 (linear) and R^2^ = 0.9618 (parabolic). In (**b**) the R^2^ = 0.9593 (parabolic fit) is for all the data, for the alloys KZ5, JN1, TT4, and TT7, R^2^ = 0.8372 (linear) and R^2^ = 0.9453 (parabolic) and for KZ5, JG3, TT4, and TT8, R^2^ = 0.9792 (linear) and R^2^ = 0.9939 (parabolic). In (**c**) the R^2^ = 0.9579 is for parabolic fit of all the data, for KZ5, JN1, TT4, and TT7, R^2^ = 0.8877 (linear) and R^2^ = 0.9435 (parabolic) and for KZ5, JG3, TT4, and TT8, R^2^ = 0.9748 (linear) and R^2^ = 0.99 (parabolic). In (**d**) for all data R^2^ = 0.8845 (parabolic), for KZ5, JN1, TT4, and TT7, R^2^ = 0.8226 (linear), R^2^ = 0.9963 (parabolic), and for KZ5, JG3, TT4, and TT8, R^2^ = 0.9988 (linear), R^2^ = 0.9997 (parabolic). In (**e**) for all data R^2^ = 0.9386 (parabolic), for KZ5, JN1, TT4, and TT7, R^2^ = 0.8226 (linear) and R^2^ = 0.9963 (parabolic), and for KZ5, JG3, TT4, and TT8, R^2^ = 0.9988 (linear) and R^2^ = 0.9997 (parabolic).

Figure 5 shows that the MACTi of the alloys TT7 and TT8 correlated with [ΔH_m_/T_m_] × [ΔH_m_^sd^/ΔH_m_^sp^]^−1^, T_m_^sd^/T_m_^sp^, T_m_^sp^, T_m_^sd^, ΔH_m_^sd^/ΔH_m_^sp^ and ΔH_m_/T_m_. The trends are in agreement with [32]. Note that in the plots of MACSi and MACTi versus the aforementioned parameters, where the data for TT5 or TT7 are included, the data always fall between that of the alloys TT4 and KZ5.

**Figure 6:**
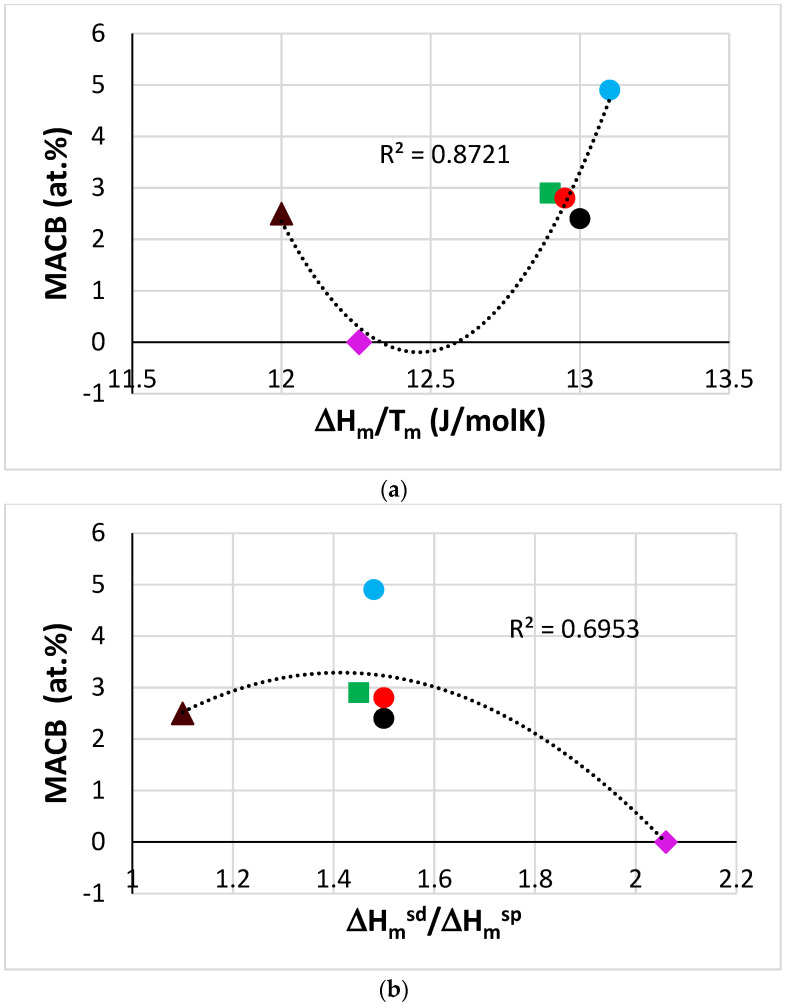

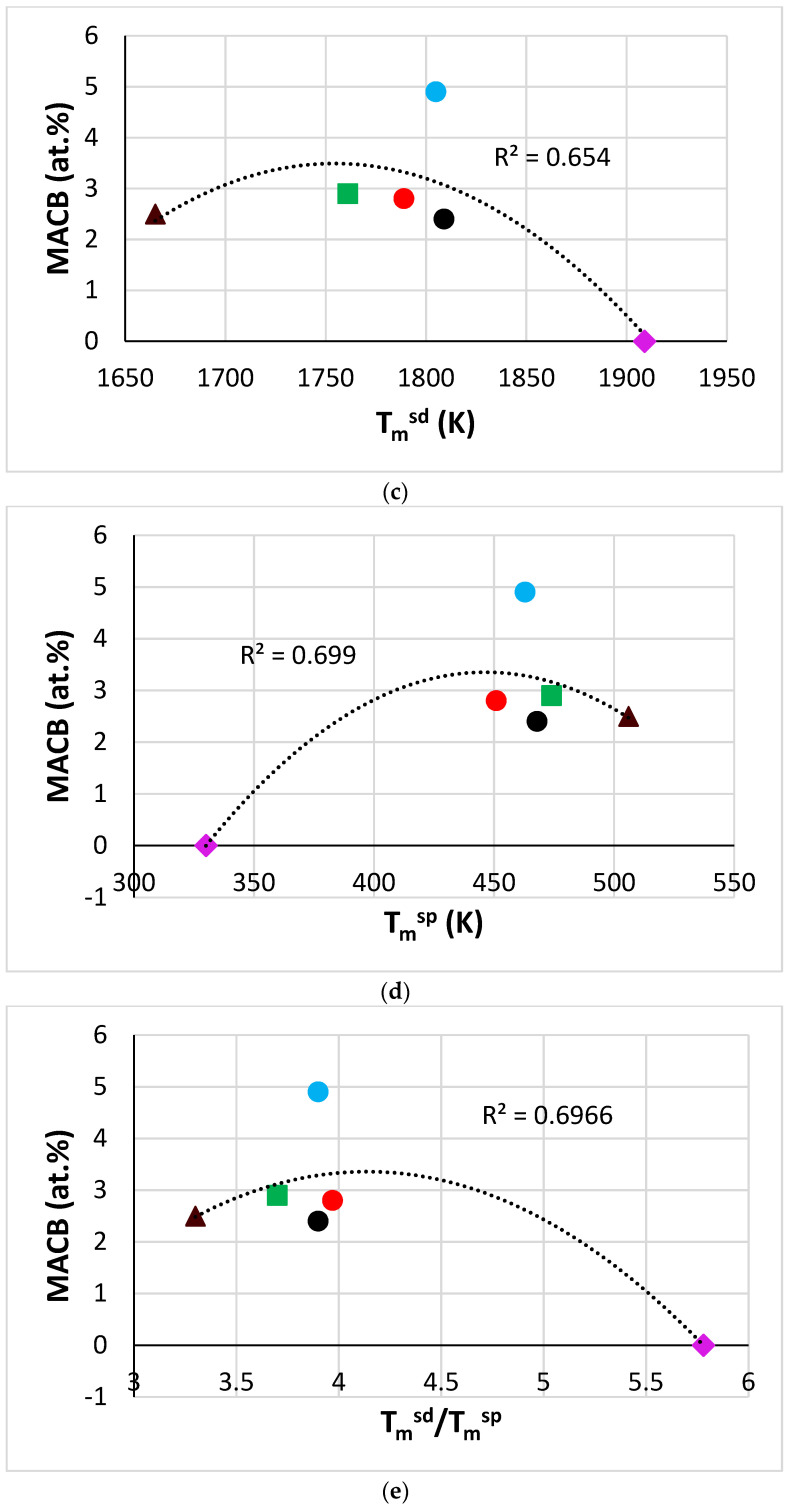

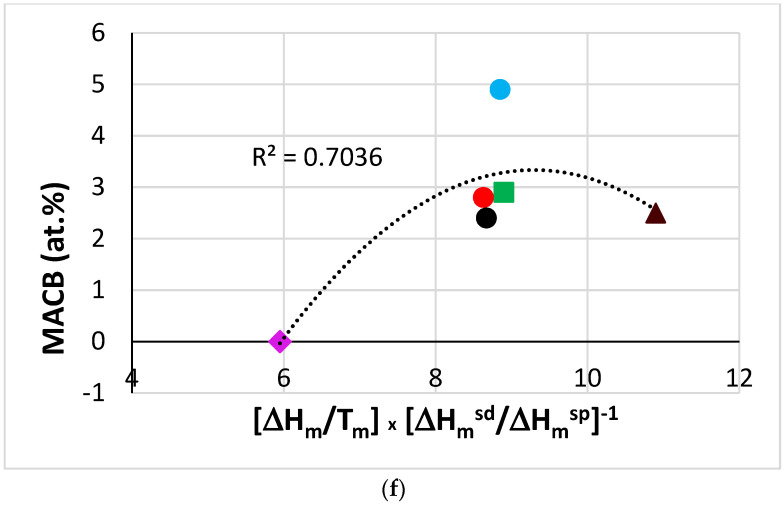
MACB versus the parameters (**a**) ΔH_m_/T_m_, (**b**) ΔH_m_^sd^/ΔH_m_^sp^, (**c**) T_m_^sd^, (**d**) T_m_^sp^, (**e**) T_m_^sd^/T_m_^sp^, and (**f**) [ΔH_m_/T_m_] × [ΔH_m_^sd^/ΔH_m_^sp^]^−1^ for the alloys TT4 to TT8, and KZ5. The parameters were calculated as described in [32]. KZ5 (diamond), TT4 (square), TT5 (black), TT6 (triangle), TT7 (red), and TT8 (blue). Parabolic fit of data excluding the alloy TT8 gives (**a**) R^2^ = 0.7426, (**b**) R^2^ = 0.9803, (**c**) R^2^ = 0.9979, (**d**) R^2^ = 0.9739, (**e**) R^2^ = 0.9752, and (**f**) R^2^ = 0.9799. In (**a**) R^2^ = 0.6875 if KZ5 were to be excluded. In (**a**) minimum ΔH_m_/T_m_ ≈ 12.45 J/molK for parabolas with R^2^ = 0.8721, R^2^ = 0.7426, and R^2^ = 0.6875, and MACB is 0 and 0.5 at.%, respectively for the latter two R^2^ values.

Data for MACB for RM(Nb)ICs where B was in synergy with/without Al and/or Cr or Mo were given in [27], where the need to study further MACB in RM(Nb)ICs using the parameters discussed in [32] was highlighted. New data for MACB were given in this paper. The data for MACB in Figure 6 show parabolic fit with maxima or minima, respectively for the parameters ΔH_m_/T_m_, ΔH_m_^sd^/ΔH_m_^sp^, T_m_^sd^, T_m_^sp^, T_m_^sd^/T_m_^sp^ and [ΔH_m_/T_m_] × [ΔH_m_^sd^/ΔH_m_^sp^]^−1^. Note that the data are for the alloys TT4 to TT8, and KZ5 (for the inclusion of KZ5 see start of the macrosegregation section). Note that in Figure 6b to f the parabolic fit of data improves significantly (R^2^ increases) when the alloy TT8, which has the highest MACB (Table 8), is excluded (see figure caption for alternative R^2^ values).

Parabolic fit of MACB versus the parameters ΔH_m_^sd^, ΔH_m_^sd^/ΔH_m_^sp^ and T_m_^sd^ was also reported for the alloys TT1, TT2, TT3, TT4, and TT8 in [27], where the increase in R^2^ values for the parabolic fit of data was noted when the alloy TT8 was excluded. Moreover, it should be noted that the plots of MACB versus the parameters ΔH_m_^sd^/ΔH_m_^sp^ and T_m_^sd^ exhibited maxima both in this work and in [27]. More data for MACB in RM(Nb)ICs, RM(Nb)ICs/RCCAs, and B containing RCCAs are required (i) to ascertain parameters and their trends for the description of MACB in metallic UHTMs and (ii) to enable the use of relationships between MACB and aforementioned parameters in NICE.

### 4.2. Microstructures

**Alloy TT5**: The microstructure of TT5 can be compared with that of the alloys TT4 [27] and KZ6 [28], see Table 4. The effects of the synergy of B with Ta on the macrosegregation of Si and Ti were discussed in the previous section. The phases in the microstructure of all parts of TT5-AC were bcc Nb_ss_, T2, and D8_8_ with some Nb_ss_ + T2 eutectic, as was the case in TT4-AC. Ti rich Nb_ss_ and Ti rich T2 were present in TT5-AC, the former was B free. Ti rich T2 also was observed in TT5-HT. The addition of Ta in TT5, similar to the addition of Ta in KZ6, did not suppress the formation of the eutectic, which is stabilised by the synergy of B, Si [37,38], and Ti [26]. Furthermore, compared with the alloy TT4, the addition of Ta did not suppress the D8_8_ silicide in TT5. This and the results for the alloys TT4 and TT8 in [27], as well as the results for TT6 and TT7 in this work, support the conclusion that it is the synergy of B, Al, and Cr that leads to the formation and stability of the D8_8_ silicide in RM(Nb)ICs and RM(Nb)ICs/RCCAs.

In the alloy KZ6, the addition of Ta did not lead to the formation of Nb_3_Si or Cr rich phase(s). Compared with TT4-AC, with the addition of Ta in TT5, the Nb_3_Si silicide and a Cr and Ti rich phase formed in certain parts of the ingot. Tantalum forms tetragonal Ta_3_Si (tP32, prototype Ti_3_P) [39] which is isomorphous with Nb_3_Si [11,39]. The Nb_3_Si was stable in TT1 [26] and was formed in the bulk of TT2-AC [27]. The data would thus suggest that the synergy of Ta with Al, B and Cr (i) promoted the formation of Nb_3_Si and (ii) decreased the sensitivity of formation of the Nb_3_Si to cooling rate, given that this silicide was formed in the top and bottom parts of the ingot of TT5-AC. Considering the chemical composition of Nb_3_Si in TT2-AC [27] and TT5-AC it is concluded that in the presence of Ta the solubility of B in Nb_3_Si increased (the Nb_3_Si in TT2-AC was B free) and the concentrations of Si and Ti decreased.

A Cr and Ti rich phase formed in the top and bulk of the ingot of TT5-AC. A Cr and Ti rich phase was observed in all parts of the ingot of TT2-AC (in which the D8_8_ was not observed [27]). The formation of the Cr rich C14-NbCr_2_ Laves phase that was observed in KZ5-AC was suppressed in KZ6-AC with the addition of Ta, whereas a Cr rich C14-NbCr_2_ Laves phase was formed in the top and bottom parts of the ingot of TT2-AC [27]. Compared with TT2-AC, the Cr and Ti rich phase in TT5-AC was leaner in Ti but richer in Si and exhibited solubility for Ta. In both TT2-AC and TT5-AC the Cr and Ti rich phase was B free. The data would suggest (a) that the partitioning of Cr in TT5-AC was affected by its synergy with Ta, Al, and B (the melt surrounding the T2 and D8_8_ silicides was Cr rich and B rich (the D8_8_ is very rich in B compared with T2, Table 5); (b) that the said synergy made the formation of the Cr and Ti rich phase sensitive to cooling rate (the phase was not formed in the bottom of the ingot where solute partitioning was affected by cooling rate); (c) that the formation of the latter phase was controlled by the partitioning of Cr rather than that of Ti; and (d) that the suppression of the formation of the C14-NbCr_2_ Laves phase was caused by the synergy of Ta with Al and Cr.

The Ti concentration in the T2 in TT5-AC was very close to that in the Ti rich T2 in the as cast alloys TT1 [26], TT2, and TT3 [27]. Furthermore, in TT5-AC the Ti concentration in the Ti rich T2 was very close to that in the very Ti rich T2 in TT3-AC and the Ti rich T2 in TT4-AC [27]. The data for the alloys TT1, TT2, and TT3 indicated that Al enhanced the partitioning of Ti in T2, and the data for TT4 suggested that the formation of the D8_8_ (which is B rich and Ti poor compared with the T2) was made certain by the synergy of B with Al, Cr, and Ti. The data for TT5 showed that the synergy of Ta with Al, B, Cr, and Ti did not affect the partitioning of the latter in T2. Thus, owing to the partitioning behaviour of Ti in T2 in TT5, the melt surrounding the T2 became lean in Ti and rich in B and from this melt formed the D8_8_ silicide.

In the Nb_ss_, as the Ti concentration increased, so did the concentration of Al and in particular that of Cr, but the opposite was the case for Ta (Table 5). The same was the case for Al, Cr, Ta, and Ti in the Nb_ss_ in KZ6-AC [28]. Compared with the latter, where the Si concentration was in the range 3.8 to 7.4 at.% [28], the concentration of Si in the Nb_ss_ and Ti rich Nb_ss_ in TT5-AC was significantly lower (Table 5) and similar to that in other B free RM(Nb)ICs and in the alloys TT1 [26] and TT2 to TT4 [27]. After the heat treatment, the Si concentration in the Nb_ss_ was below 1 at.%, also in agreement with other B free RM(Nb)ICs (e.g., see [34]) and the alloys TT1 [26] and TT2 to TT4 [27] but not with KZ6-HT, where it was in the range 2.8–5.5 at.%. The data would suggest that the solubility of Si in the Nb_ss_ can be controlled by the synergy of Al, Cr, and Ti with Ta or Ta and B. The B concentration in the Nb_ss_ in TT5-AC decreased with increasing Ti content, in agreement with the Nb_ss_ in the as cast alloys TT1 to TT4, and increased significantly in TT5-HT (Table 5), which was not the case in the heat-treated alloys TT1 [26] and TT2, TT3, and TT4 [27].

**Alloy TT6**: The results for the alloy TT6 can be compared with the data for the alloys TT4 [27] and ZX8 [29], see Table 4. The comparison allows us to understand how the synergy of B and Sn affects the formation of phases and their stability in RM(Nb)ICs/RCCAs in the presence of Al and Cr. The effect of the above-mentioned synergy on MACSi and MACTi was discussed in the previous section. The said synergy induced macrosegregation of Cr (Table 3) compared with TT4-AC, where MACCr was 1.4 at.% [27], but reduced it compared with ZX8, where MACCr = 4.7 at.% [29]. In other words, the simultaneous addition of B and Sn in TT6 resulted to a significantly more chemically homogeneous microstructure, compared with ZX8-AC.

In TT6-AC three silicides were formed, namely, the Nb_3_Si, T2, and D8_8_ (Table 4), the T2 owing to the synergy of B with Ti and Si [26] and the D8_8_ because of the synergy of B with Al, Cr, Si, and Ti [27]. The T2 and D8_8_ silicides were stable in TT6, similarly to TT4 but not the Nb_3_Si. In other words, with the addition of Sn, the Nb_3_Si formed in TT6-AC (the Nb_3_Si is stabilised by the synergy of B with Si and Ti [26] but not by the synergy of B with Al, Si, and Ti [27]) but was not stable (absent in TT6-HT). Given that Sn in B free RM(Nb)ICs with or without Ti addition suppresses the Nb_3_Si [29,36,40], it is concluded that the synergy of Al, B, Cr, Si, and Ti with Sn cannot (i) stabilize the Nb_3_Si and (ii) destabilise the D8_8_. The structure of the stable silicides in TT6 did not change after the heat treatment, differently from ZX8-HT where the high temperature tetragonal βNb_5_Si_3_ (tI32 W_5_Si_3_-type, D8_m_) transformed to the low temperature tetragonal αNb_5_Si_3_.

The synergy of B and Sn with Al, Cr, Si, and Ti also (a) decreased the vol.% of Nb_ss_ in TT6-AC, compared with TT4-AC, but did not suppress its formation, differently from ZX8-AC where the solid solution was not observed (Table 4, [29]); (b) decreased significantly the vol.% of the Nb_ss_ + T2 eutectic, owing to the formation of Nb_3_Sn; (c) had an effect on the chemical composition of the Nb_ss_, which was rich in Ti (Nb/Ti ≈ 0.6 in TT6-AC) and B free (Table 4 and Table 5); (d) did not stabilise the Nb_ss_ (absent in TT6-HT), which was stable in ZX8-HT; (e) destabilised the C14-NbCr_2_ Laves phase, which was stable in ZX8 [29]; and (f) stabilised the A15-Nb_3_Sn (owing to the synergy of Sn with Si and Nb [40]), the vol.% of which increased after the heat treatment (Table 2), similarly to the alloys NV6 and NV9 [40]. It should be noted that the synergy of B and Sn can control the vol.% and the stability of Nb_ss_ (as does the synergy of B and Hf regarding the vol.% of D8_8_, see below).

Comparison of the data for the alloys ZX7 [41] and ZX8 [29], where Al, Cr, Si, and Ti were in synergy with Sn (at nominal Sn concentration of 2 and 5 at.%, respectively), showed: (a) that the formation of Nb_ss_ and Nb_ss_ + βNb_5_Si_3_ eutectic in the cast alloys (present in ZX7-AC) and (b) the stability of A15-Nb_3_Sn (absent in ZX7-AC, present in ZX7-HT) depended on the concentration of Sn. However, this was not the case regarding the formation and stability of the C14-NbCr_2_ Laves phase (present in ZX7-AC, ZX7-HT, ZX8-AC, and ZX8-HT). In the alloy TT6 the average Sn concentration, even though was lower than the nominal one, was not low enough to ensure the formation of Nb_ss_ and Nb_ss_ + T2 eutectic. Thus, the absence of the Laves phase in TT6 must be linked with solute partitioning between T2, D8_8_ (Cr content similar to that in βNb_5_Si_3_), Nb_3_Si, and Nb_3_Sn (Cr content similar in the two intermetallics) and Nb_ss_ (higher Cr content owing to the solid solution being Ti rich) that did not increase the concentration of Cr in the melt in between the silicides to levels high enough to ensure Laves phase formation.

The Nb_ss_ in TT6-AC and ZX8-HT had Cr/Al ≈ 1.5. In TT6-AC the Nb_ss_ had Si + Sn = 5 at% and Si/Sn = 0.22, close to the values in NV9-AC (5.7 at.% and 0.3, respectively) and to the Si + Sn sum in ZX7-AC (5.1 at.% [41]) and the Si/Sn ratio in ZX8-HT (0.3). In Table 9 are compared the Si + Sn, Si + Sn + Al, and Si + Sn + Al + B concentrations and the Si/Sn, (Si + Al)/Sn, and (Si + Al + B)/Sn ratios in A15-Nb_3_Sn in the AC and HT alloys TT6, ZX8, [29] and NV9 [40]. The data show: (i) that the Si + Sn, Si + Sn + Al, and Si + Sn + Al + B concentrations increased with the addition of Al and B but did not exceed 25 at.%, in agreement with the composition range of Nb_3_Sn [39]; (ii) that the Si + Sn + Al content in Nb_3_Sn in TT6 was similar to that in ZX8 and about 19.8 at.%; and (iii) that Al and B substitute for Si in the A15 compound in which the aforementioned ratios were around one.

**Alloy TT7:** The microstructure of TT7 will be compared with that of the alloys TT4 [27] and JN1 [30] to help us understand how Hf affects the formation and stability of phases in RM(Nb)ICs/RCCAs when it is in synergy with Al, B, Cr, Si, and Ti. The effect of the said synergy on MACSi and MACTi was discussed in Section 4.1. Similar to Sn in TT6 and compared with TT4-AC, the above-mentioned synergy increased the macrosegregation of Cr (Table 3). The characterisation of the microstructure was difficult owing to the partitioning of Hf, even though the macrosegregation of B, Cr, Si, and Ti was low compared with other alloys (see Table 3 and Table 8). It should be noted that Hf partitions to both Nb_ss_ and 5-3 silicides where “it follows” Ti, meaning the Ti rich phases are also Hf rich. Take notice of the fact that the synergy of Hf with Sn in B and Ti free RM(Nb)ICs also makes difficult microstructure characterisation [36].

Solid solution and 5-3 silicides were present in all three alloys JN1-AC [30], TT4-AC [27], and TT7-AC (Table 4), eutectic only in the former two, and Nb_3_Si only in the TT7. The synergy of Hf with B in TT7 not only suppressed the Nb_ss_ + T2 eutectic but also significantly reduced the vol.% of D8_8_. The said synergy also affected the chemical composition of the Nb_ss_. Indeed, compared with TT4-AC and JN1-AC, with the simultaneous addition of B and Hf in TT7 the solid solution became poorer in Al, and in the Ti rich Nb_ss_, the Ti and Cr concentrations decreased compared with TT4-AC and increased compared with JN1-AC, whereas the Hf content was slightly lower compared with JN1-AC. Furthermore, differently from TT5 and TT6, the Ti rich Nb_ss_ was not B free.

Compared with TT4-AC, the addition of Hf did not have a significant effect on the concentration of B in T2, or Ti rich T2 and D8_8_ in TT7-AC. This would suggest that the addition of Hf did not affect the partitioning of B to T2 and D8_8_ during solidification. After the heat treatment, the average concentration of B in the T2 and Ti rich T2 did not change significantly, similarly to the T2 in TT4-HT, and the same was the case for the B concentration in the D8_8_ silicide.

The Nb_3_Si was not observed in the bottom of the ingot of TT7-AC, which would suggest that its formation was not possible during solidification under high cooling rate conditions. The Nb_3_Si is the primary phase in the solidification of Nb-19Si-5Hf [42,43] and was formed in the as cast Hf containing alloys YG1 and YG3 [44] but not in YG2 [44] (for the nominal compositions of the alloys see Appendix A). Furthermore, Hf and Si do not form a 3-1 silicide [39]. Thus, the data show that the Nb_3_Si cannot form when Nb and Si, in the absence of Ti, are in synergy with Al and Hf (the case of YG2) and can form when Nb and Si are in synergy with Ti and Hf (the case of YG3) or with Ti, B, and Cr (the case of TT2 [27]) but cannot form when Nb and Si are in synergy with Ti, B, and Al (the case of TT3 [27]), or Ti, B, Al, and Cr (the case of TT4 [27]). It is suggested that in TT7-AC the formation of Nb_3_Si was made possible by the synergy of Hf with B and Cr, which was effective in overcoming the effect of Al. The latter synergy is also suggested to control the sensitivity of the formation of Nb_3_Si to cooling rate (the Nb_3_Si was not observed in the bottom of the ingot of YG1-AC [44]).

**Contamination by interstitials**: In all three alloys of this study, contamination by nitrogen was not observed after the heat treatment, meaning TiN was not formed in their microstructures. Furthermore, hafnia was not observed in the as cast and heat-treated alloy TT7. The data for the alloys TT1 [26], TT2, TT3, TT4, and TT8 [27] and TT5 to TT7 indicate: (a) that the synergy of B with Al “keeps under control” contamination by nitrogen (compare TT1-HT, TT2-HT with TT3-HT); (b) that when B and Al are in synergy with Cr, the Al cancels the opposing effect of Cr (compare TT2-HT with TT4-HT); (c) that the synergy of Cr with Mo overrules the said effect of the synergy of B with Al (compare TT4-HT with TT8-HT), whereas the synergy of Ta with Cr does not (compare TT8-HT with TT5-HT); and (d) that the synergy of B and Al with Sn or Hf enhances the beneficial effect of the synergy of the former two elements (compare TT6-HT and TT7-HT with TT4-HT and TT8-HT).

**Solid solution:** In TT5-AC, TT7-AC and TT5-HT there were B free Nb_ss_ grains. The Ti rich Nb_ss_ also was B free in TT5-AC, TT6-AC and TT7-AC. Furthermore, B free solid solution was observed in TT8-AC and TT8-HT [27]. In all the aforementioned alloys, the said grains were not Si free, with the exception of the Nb_ss_ in TT7-HT, which contained B. Silicon free solid solution can form in RM(Nb)ICs and RM(Nb)ICs/RCCAs [1,3,4,12] with RM = Mo, W [12,21,45] or RM = Mo, Ta [46] or RM = Ta, W [24] additions but not in RM(Nb)ICs where RM = Ta [28]. In such alloys, the parameter δ_ss_ of the Si free solid solution is less than about 5 [1,3,4,12]. Thus, this work, and [27], would suggest that the synergy of B with Ta or Mo in RM(Nb)ICs and RM(Nb)ICs/RCCAs can lead to the formation of B free and Si containing solid solutions, whereas the synergy of B with Hf could possibly encourage the formation of Si free and B containing Nb_ss_. The presence or absence of B and Si in bcc Nb_ss_ is expected to have an effect on its mechanical properties and oxidation.

**Figure 7:**
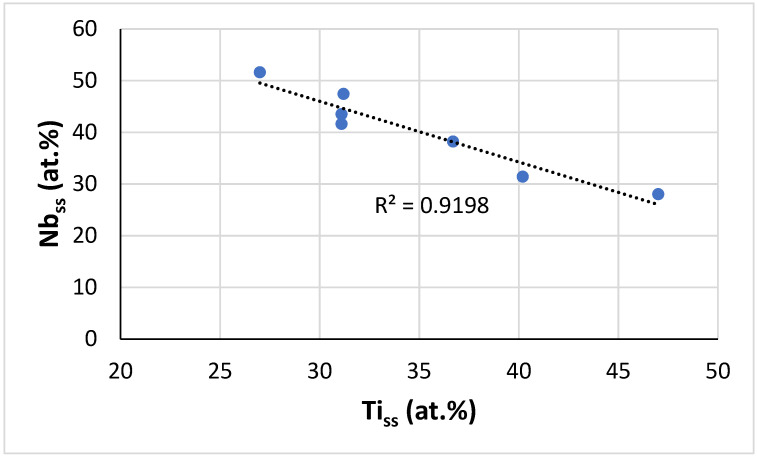
Nb versus Ti concentration in bcc Nb_ss_ in the alloys TT5, TT6, and TT7.

In the solid solution, the B or Nb content decreased with increasing Ti concentration (Figure 7), whereas the Si concentration increased (figure not shown). NICE suggests that in B containing RM(Nb)ICs/RCCAs with one other RM in addition to Nb, the bcc solid solution could be: (a) Si free for Ti ≈ 30 at.%, for which its B, Al, and Cr concentrations would be about 3, 6.5, and 10 at.%, respectively, in reasonable agreement with the data for the Nb_ss_ in TT7-HT; and (b) B free for Ti ≈ 40 at.%, for which its Si, Al, and Cr concentrations would be about 1.3, 7.8, and 16 at.%, respectively, in slightly better agreement with the data for TT5-AC but not with the data for TT6-AC. Further research is essential to ascertain whether B and Si free bcc solid solution can form in B containing metallic UHTMs with RM = Mo,W or Ta,W or Mo,Ta simultaneous additions and Ti/Si higher or less than 1 [1,21,24].

**Figure 8:**
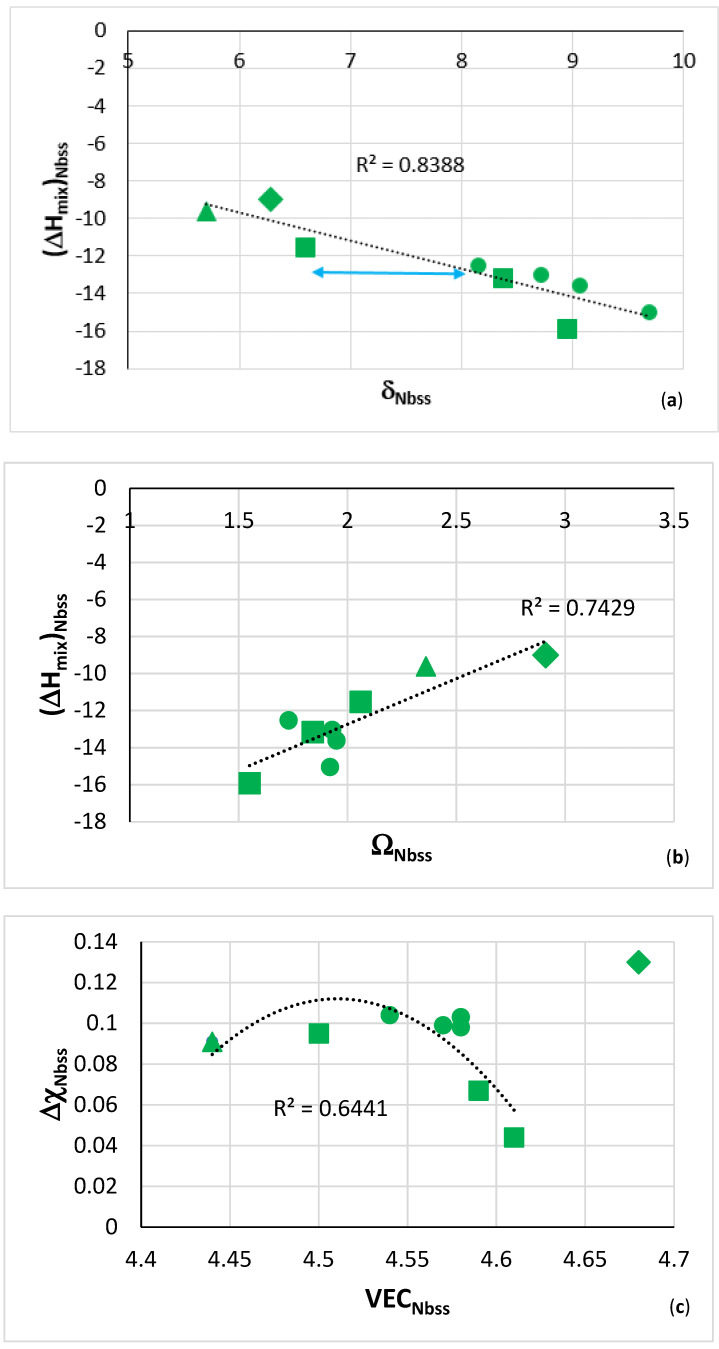
(**a**) ΔH_mix_ versus δ, (**b**) ΔH_mix_ versus Ω, and (**c**) Δχ versus VEC of bcc solid solutions in the B containing alloys TT4 to TT8. TT4 squares, TT8 diamond, TT6 triangle. In (**a**) the gap in δ values is shown by blue arrows. The parameters were calculated as described in [12].

It should be noted (a) that the Ti rich Nb_ss_ in TT4 and TT8 [27], and TT5 and TT7 (Table 5) could be considered as bcc “RCCA phase” owing to its chemical composition and (b) that the parameters of the solid solution in the alloys TT4 to TT8 exhibit trends typical of those observed in the bcc solid solutions of RM(Nb)ICs and HEAs, see Figure 8. The solid solutions in these alloys have δ higher than 5, and Δχ less than 0.130, meaning that their parameter Δχ is outside the gap of Δχ values for bcc Nb_ss_ in RM(Nb)ICs [1,3,4,12]. There is also a gap in δ parameter values between 6.6 and 8.1 (Figure 8a). Note, the opposite trends exhibited by the parameters δ and Ω versus the parameter ΔH_mix_. Moreover, note that with the exception of TT8, the Δχ_Nbss_ in the other alloys is less than 0.105 (Figure 8c). Note that (a) above is not uncommon in RM(Nb)ICs, for example, see [47].

**D8_8_ and T2 silicides**: In the alloys of this study, as well as in the alloys TT4 and TT8 [27], the Nb_ss_ was in equilibrium with the T2 and D8_8_ silicides, whereas in alloys where B was in synergy with Al or Cr individually, i.e., the alloys TT2 and TT3 [27], rather than simultaneously, as in the alloys TT4 to TT8, it was in equilibrium only with the T2 silicide. The D8_8_ and T2 silicides had: distinct (a) average Si/B ratios, respectively 0.5 and 5, compared with 0.5 and 4 in TT4 and TT8 [27]; and (b) average <Nb>/<Si> ratios, respectively 1.47 and 1.65, compared with 1.4 and 1.63 for TT4 and TT8, where <Nb> = Nb + TM + RM, TM = Cr, Hf, Ti, RM = Mo, Ta and <Si> = Si + B + Al + Sn (Table 6 and [27]). The D8_8_ had average Si + B = 40.4 at.% compared with 41.5 at.% for the alloys TT4 and TT8 [27], whereas the T2 had average Si + B, Si + B + Al and Si + B + Al + Sn sums, respectively 34.5 at.%, 37.5 at.%, and 38.2 at.% (or 37.9 at.%, the average of the latter two values) (Table 6), compared with 34.8 at.% and 38 at.% for the alloys TT4 and TT8 [27].

The Nb_ss_ and the tetragonal D8_l_ (αNb_5_Si_3_) and hexagonal D8_8_ 5-3 silicides were formed in the microstructures of the alloys (nominal compositions, at.%) AC6 (Nb-24Ti-18Si-2Al-2Cr-2Hf-6B) and AC7 (Nb-24Ti-18Si-2Al-4Cr-2Hf-4B) in [9], in agreement with TT7, whereas the microstructures of the B free Nb-24Ti-18Si-2Hf based alloys with Cr = 4 at.% and Al = 4 or 6 at.% consisted of Nb_ss_ and tetragonal D8_m_ 5-3 silicide (i.e., βNb_5_Si_3_), in agreement with JN1 (Table 4). For the microstructure of the alloy Nb-22Ti-16Si-5Cr-4Hf-3Al-5B (nominal composition, at.%) Zhang and Guo [8] reported the Nb_ss_, hexagonal (D8_8_) 5-3 silicide, eutectic of the two phases and Nb_3_Si. When the alloy Nb-22Ti-16Si-6Cr-4Hf-3Al-1.5B-0.006Y (nominal, at.%) was cast using directional solidification (DS) [7], Nb_ss_ and Nb_ss_ + Nb_5_Si_3_ eutectic were reported for the majority of the microstructure, but, depending on the withdrawal rate (R) in DS, in some of the eutectic cells Ti and Cr rich Nb_ss_ was observed together with NbCr_2_ Laves phase and for R = 2.5 μm/s the tetragonal (D8_m_) and hexagonal (D8_8_) 5-3 silicides were formed. Note that none of the aforementioned studies [7,8,9] reported the presence of Nb_3_B_2_. The same was the case for the alloy Nb-22Ti-16Si-3Al-6Cr-4Hf-5B (nominal composition, alloy N4 in [48]) in which the Nb_ss_, Nb_3_Si and tetragonal and hexagonal 5-3 silicides were observed in the 0.2 kg cast button/ingot.

**Figure 9:**
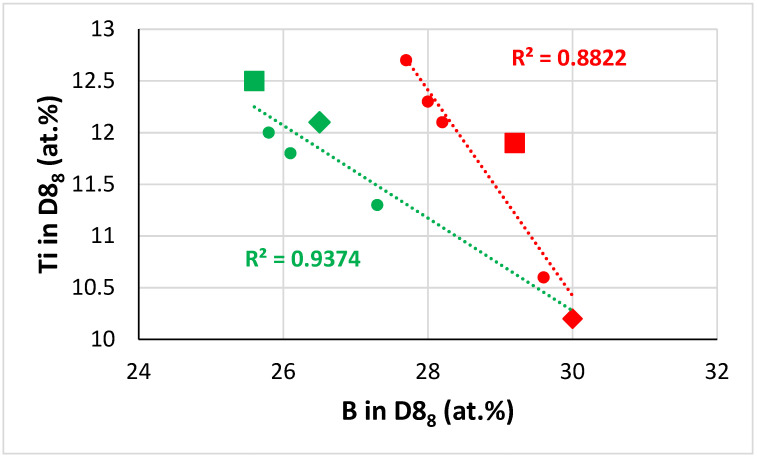
Ti versus B in the D8_8_ silicide in the alloys of this work and the alloys TT8 (diamonds) and TT4 (square).

**Figure 10:**
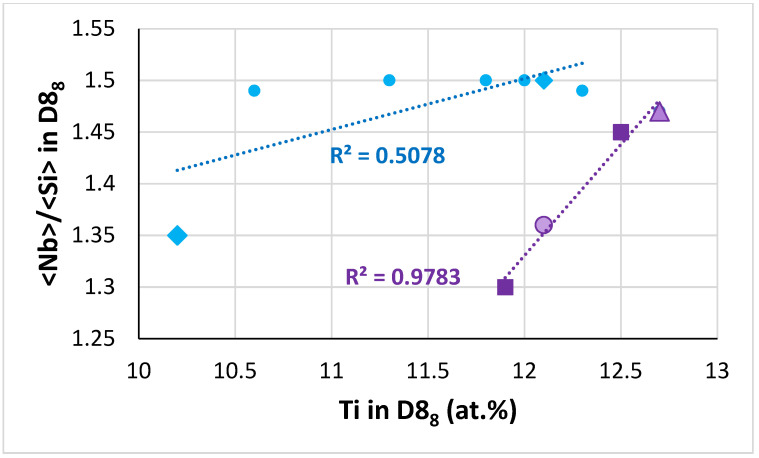
<Nb>/<Si> ratio versus Ti in the D8_8_ silicide for the alloys of this study and the alloys TT8 (diamonds) and TT4 (squares). Light purple triangle for TT7-AC and light purple circle for TT6-AC. R^2^ = 0.8351 for the blue data fitted to parabolic regression.

**Figure 11:**
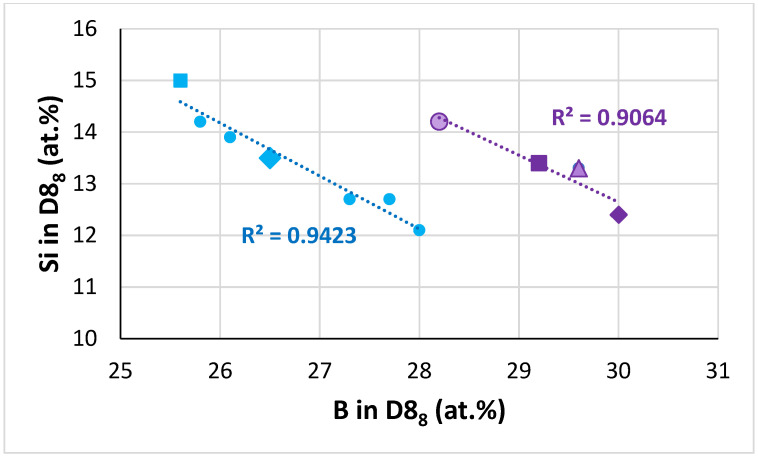
Si versus B in the D8_8_ silicide of the alloys of this work and the alloys TT8 (diamonds) and TT4 (squares). Light purple triangle for TT7-AC and light purple circle for TT6-AC.

**The D8_8_ silicide:** In the D8_8_ silicide in the alloys TT4 to TT8, as the B concertation increased the Ti content decreased (Figure 9). In Figure 9, the data converge to B and Ti about 30.3 and 10.16 at.%, respectively. As the Ti concentration in the D8_8_ silicide increased so did the <Nb>/<Si> ratio, see Figure 10. In this figure, when the data are fitted to linear regressions, the two lines cross at Ti in D8_8_ about 13 at.% or <Nb>/<Si> in D8_8_ about 1.55, whereas the two lines cross at Ti and <Nb>/<Si> in D8_8_, respectively about 12.5 at.% and 1.45, when one is fitted with linear regression and the other with parabolic regression, see figure caption. For Ti in D8_8_ equal to 13 at.%, Figure 9 gives B 23.9 at.% and 27.4 at.%. For Ti in D8_8_ equal to 12.5 at.%, Figure 9 gives B equal to 25.6 at.% and 27.9 at.%. It is suggested that the D8_8_ has 10.2 < Ti < 13 at.%, 23.9 < B < 30 at.% and <Nb>/<Si> less than 1.55, which is consistent with Table 6. Furthermore, in the D8_8_ silicide the Si concentration decreased as the B content increased, see Figure 11. In Figure 11, the data fall in two groups with essentially the same slop, consistent with the Si/B ratio being constant (Table 6).

**Figure 12:**
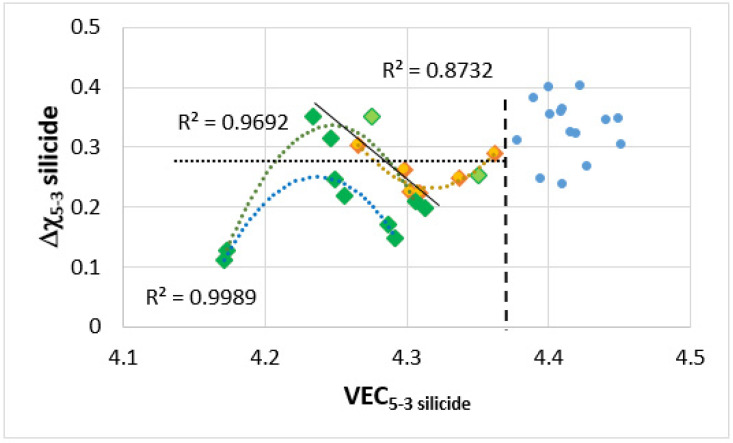
Δχ versus VEC map of tetragonal T2 and Nb_5_Si_3_ in the B free RM(Nb)ICs alloys KZ5, KZ6, JN1, ZX8, JG3, the B containing RM(Nb)ICs TT4, TT8, and RM(Nb)ICs/RCCAs TT5 to TT7. The B containing alloys are shown by diamonds; green diamonds for the RM(Nb)ICs/RCCAs TT5, TT6, and TT7 and gold diamonds for the RM(Nb)ICs TT4 and TT8. The B free alloys are shown by blue circles. For the dotted horizontal line see text. The parameters were calculated as described in [14].

**The T2 silicide**: The Δχ versus VEC map in Figure 12 shows data for the tetragonal T2 and tetragonal Nb_5_Si_3_ in the alloys TT4 to TT8 and KZ5, KZ6, JN1, ZX8, and JG3 (see Appendix A for nominal compositions). In the B containing alloys the silicide had VEC less than about 4.36 (shown by the black vertical dashed line) and occupied a distinct different area in the map in agreement with previous research for RM(Nb)ICs and RCCAs [1,3,4]. In Figure 12, the B containing alloys are shown with diamonds; green diamonds show the RM(Nb)ICs/RCCAs TT5, TT6, and TT7 and gold diamonds the RM(Nb)ICs TT4 and TT8. In Figure 12, the B free alloys are shown with blue filled circles. Note: (i) the parabolic fit of the data for TT4, TT8 with R^2^ = 0.8732; (ii) the data points for the T2 in TT5-AC (light green diamonds); (iii) the data for TT6 with R^2^ = 0.9989 and for TT7 with R^2^ = 0.9692; (iv) the opposite trends of the parabolas for the RM(Nb)ICs/RCCAs compared with the RM(Nb)ICs, and that the parabolic fit of the data for TT5 is poor but shows the same trend as for the silicides in the alloys TT6 and TT7; and (v) that the data for T2 in RM(Nb)ICs/RCCAs TT5-HT, TT6, and TT7 lie to the left of the thin black line running at about 45 degrees from left to right.

**Figure 13:**
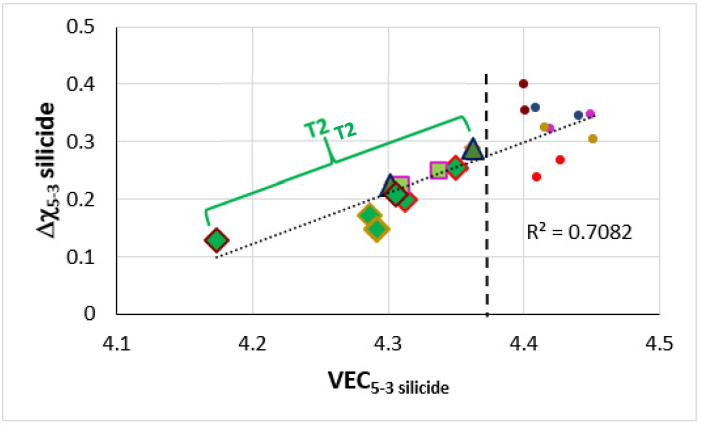
Δχ versus VEC map for the tetragonal T2 and Nb_5_Si_3_ excluding the data for Ti rich T2 and Ti rich Nb_5_Si_3_ in the B free RM(Nb)ICs alloys KZ5, KZ6, JN1, ZX8, JG3, the B containing RM(Nb)ICs TT4, TT8 and RM(Nb)ICs/RCCAs TT5 to TT7. The parameters were calculated as described in [14].

The Δχ versus VEC map for the tetragonal T2 and Nb_5_Si_3_, excluding the data for Ti rich T2 and Ti rich Nb_5_Si_3_ in the alloys TT4 to TT8 and KZ5, KZ6, JN1, ZX8, and JG3 (see Appendix A for the nominal compositions), shows linear fit with R^2^ = 0.7082 (Figure 13). The B containing alloys occupy a distinct different area in the map in agreement with previous research for RM(Nb)ICs and RCCAs [1,3,4]. The T2 has VEC and Δχ, respectively less than approximately 4.36 (vertical dashed line, Figure 12) and 0.28 (horizontal dotted line in Figure 12). In Figure 13, the green colour is used for the B containing alloys, light green squares for TT4, dark green triangles for TT8, filled circles are for the B free alloys with KZ6 red, KZ5 pink, ZX8 gold, JN1 dark red, and JG3 dark blue. When the alloys are considered in four groups, namely, (i) KZ5, JG3, TT4, and TT8; (ii) KZ5, ZX8, TT4, and TT6; (iii) KZ5, JN1, TT4, and TT7; and (iv) KZ5, KZ6, TT4, and TT5, the linear fit of the data gives R^2^ equal to 0.9167, 0.8883, 0.8493, and 0.6388, respectively.

**Figure 14:**
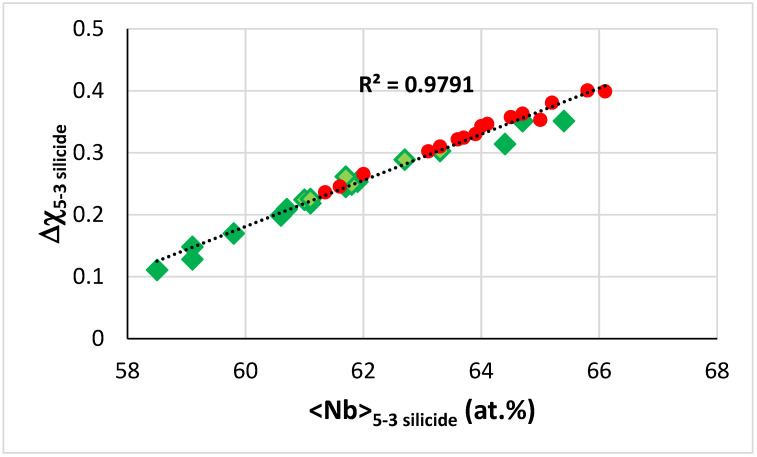
Δχ versus <Nb> for tetragonal T2 and Nb_5_Si_3_ in the RM(Nb)ICs alloys KZ5, KZ6, JN1, ZX8, JG3, the RM(Nb)ICs TT4 and TT8 and the RM(Nb)ICs/RCCAs TT5 to TT7. The B containing alloys are shown by diamonds, the B free alloys by red filled circles. The RM(Nb)ICs TT4 and TT8 are shown by light green diamonds. The parameter Δχ was calculated as described in [14].

The map of Δχ versus <Nb> for T2 and Nb_5_Si_3_ in the B free alloys KZ5, KZ6, JN1, ZX8, and JG3 (see Appendix A for the nominal compositions), and the B containing alloys TT4 to TT8 shows very good linear fit of data with R^2^ = 0.9791 (Figure 14). Note that the data for RM(Nb)ICs and RM(Nb)ICs/RCCAs overlap, which is not the case in the equivalent map of the alloys TT2, TT3, TT4, and TT8 with their reference alloys KZ4, KZ5, KZ7, and JG3.

**Figure 15:**
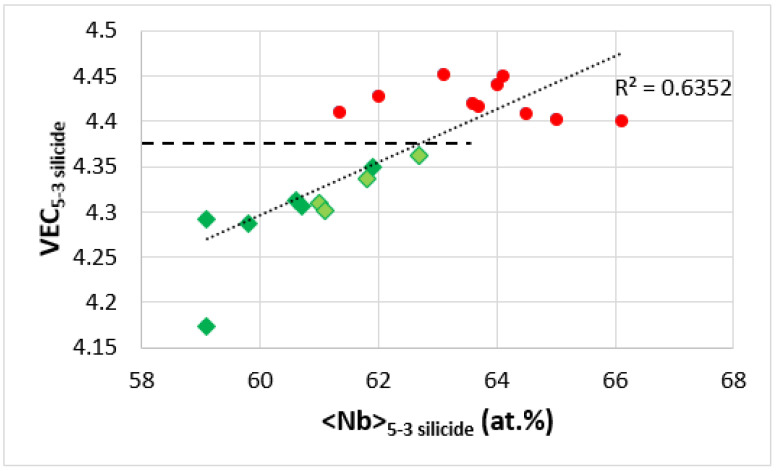
VEC versus <Nb> map for tetragonal T2 and Nb_5_Si_3_ in the B free RM(Nb)ICs alloys KZ5, KZ6, JN1, ZX8, JG3, the B containing RM(Nb)ICs TT4 and TT8 and the RM(Nb)ICs/RCCAs TT5 to TT7. B containing alloys are shown by diamonds, B free alloys by red filled circles. The RM(Nb)ICs TT4 and TT8 are shown by light green diamonds. The parameter VEC was calculated as described in [14].

The VEC versus <Nb> map for T2 and Nb_5_Si_3_ in the alloys KZ5, KZ6, JN1, ZX8, JG3, and the alloys TT4 to TT8 shows linear fit of the data with R^2^ = 0.6352 (Figure 15). Note that the data for RM(Nb)ICs and RM(Nb)ICs/RCCAs does not overlap, which also is the case in the equivalent map of the alloys TT2, TT3, TT4 and TT8 with their reference alloys KZ4, KZ5, KZ7, and JG3. When the alloys are considered in four groups, namely, (i) KZ5, JG3, TT4, and TT8; (ii) KZ5, ZX8, TT4, and TT6; (iii) KZ5, JN1, TT4, and TT7; and (iv) KZ5, KZ6, TT4, and TT5, the linear fit of the data give R^2^ equal to 0.9056, 0.8849, 0.7615, and 0.5978, respectively. The horizontal dashed line in Figure 15 shows that the T2 in the B containing alloys has VEC less than approximately 4.36 (see Figure 12 and Figure 13).

**Nb_3_Si silicide**: The Nb_3_Si was observed in the as cast alloys of this study and exhibited solubility for B, and Hf, Sn, or Ta. In all three alloys the Nb_3_Si was not stable after the heat treatment. Its B and Al contents did not vary significantly, and respectively increased and decreased from TT5 to TT7. Its Si content was essentially the same in the alloys TT5 and TT6, but significantly lower in TT7 (Si/B and Al/B ratios equal to 16, 13.9, and 6.9, and 3.6, 2.9, and 1.9, respectively in TT5, TT6 and TT7). In the alloys TT5 and TT6, the Nb_3_Si was Ti rich (Nb/Ti = 0.7) and Ti poor in TT7 (Nb/Ti = 1.6). Thus, the alloys TT5 and TT6 had <Nb>/<Si> = 3 (Si + B + Al + Sn equal to 24.7 and 24.9 at.%) but the alloy TT7 had <Nb>/<Si> = 4.7 (Si + B + Al = 17.6), which would suggest that the Nb_3_Si was the metastable Nb_3_Si-m, according to Schlesinger et al. [11] (<Nb> = Nb + TM + RM, TM = Cr, Hf, Ti, RM = Ta, <Si> = Si + B + Al + Sn).

**Figure 16:**
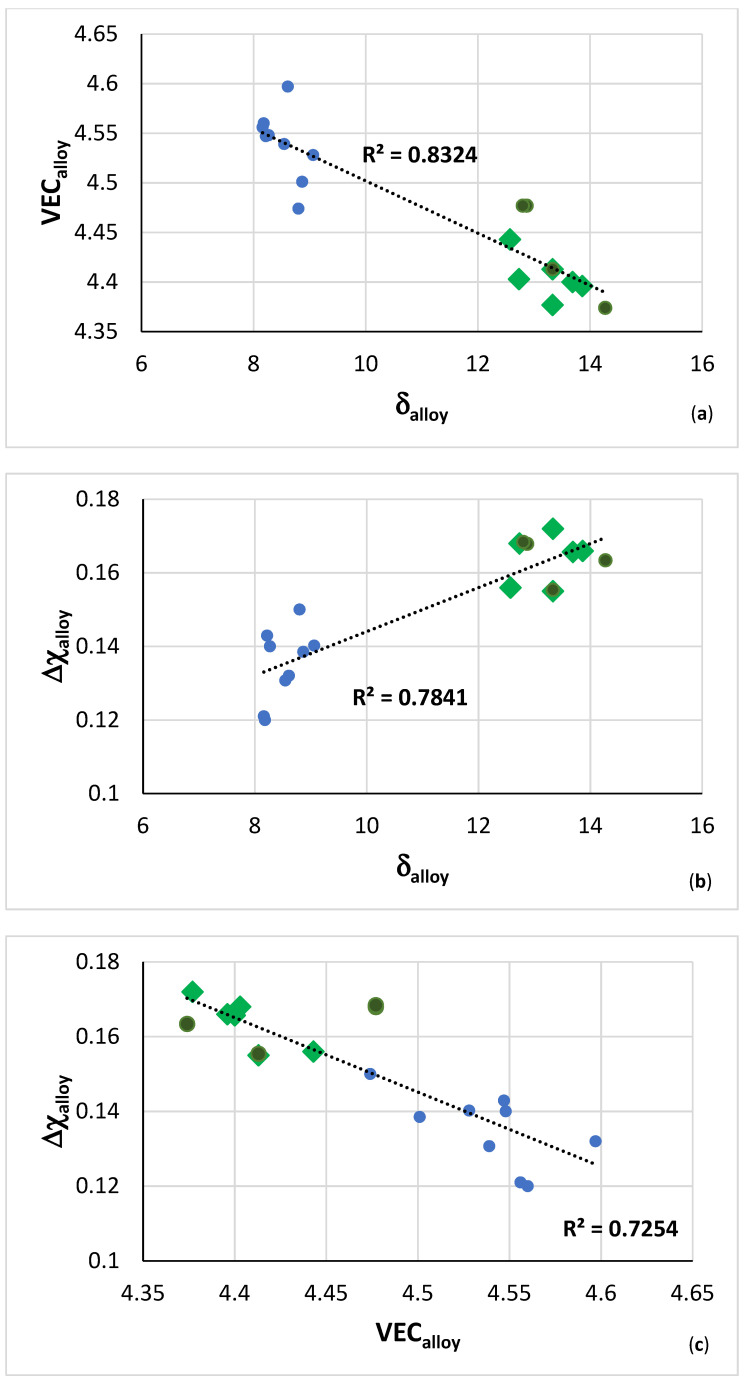
(**a**) VEC versus δ, (**b**) Δχ versus δ and (**c**) Δχ versus VEC maps for the B free RM(Nb)ICs alloys KZ5, KZ6, JG3, JN1, ZX8 (blue colour), and the B containing (green colour) RM(Nb)ICs TT4 and TT8 (green circles) and RM(Nb)ICs/RCCAs TT5 to TT7 (green diamonds). The parameters were calculated as described in [13].

**Maps of alloy parameters**: The VEC versus δ, Δχ versus δ, and Δχ versus VEC alloy maps in Figure 16 show that the B containing RM(Nb)IC and RM(Nb)IC/RCCA alloys occupy a distinct separate area in each map, in agreement with [1,3,4,13]. With the addition of B, the parameters VEC and δ, respectively decrease and increase, which, according to NICE, indicates that the alloying with B should improve oxidation, whereas the parameter Δχ increases, which, according to NICE, hints that creep could improve with alloying with B. Furthermore, the Δχ versus δ map shows that alloying with B changes both parameters in the desirable direction for creep and oxidation, meaning that, according to NICE, it should be possible to get a balance of oxidation and creep properties with alloying with B. However, the alloy designer should take into account the worse creep of the alloyed with B Nb_5_Si_3_ compared with the unalloyed silicide [3,14].

### 4.3. Properties

#### 4.3.1. Hardness and Specific Yield Strength

**Figure 17:**
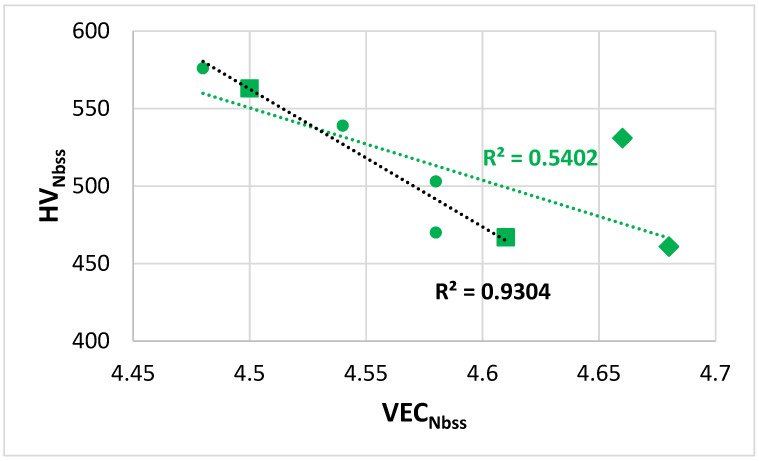
Hardness of Nb_ss_ in AC and HT RM(Nb)ICs TT4 and TT8 [27] and RM(Nb)ICs/RCCAs TT5 and TT7. All data R^2^ = 0.5402, alloys TT4, TT5 and TT7, R^2^ = 0.9304. TT4 squares, TT8 diamonds. The parameter VEC was calculated as described in [12].

The hardness of the Nb_ss_ decreased with increasing VEC_ss_, see Figure 17, a trend that is in agreement with the data in [27]. The hardness of A15-Nb_3_Sn in TT6 in the AC and HT conditions (respectively 758 and 678 HV) was less than 800 HV and within the range of values for alloyed A15-Nb_3_X in B free RM(Nb)ICs [16].

**Figure 18:**
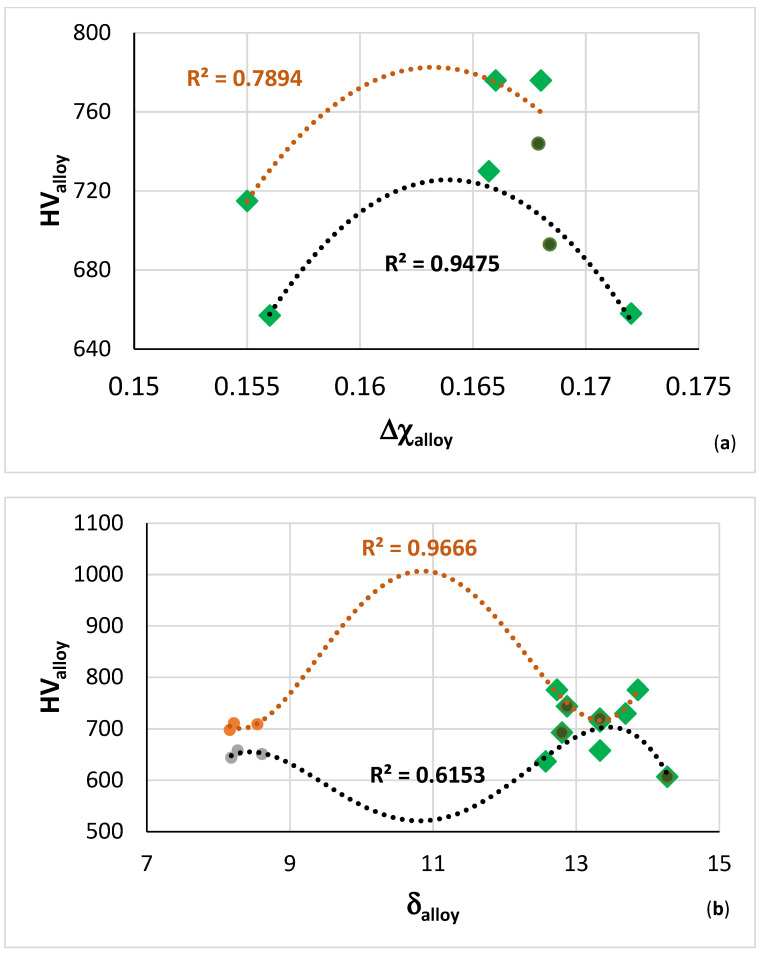
(**a**) Hardness versus Δχ map of the RM(Nb)ICs/RCCAs alloys TT5 to TT7 (diamonds) and RM(Nb)IC TT8 (circles). AC alloys R^2^ = 0.7894 and HT alloys R^2^ = 0.9475. (**b**) Hardness versus δ map of the RM(Nb)ICs/RCCAs alloys TT5 to TT7 (green diamonds), and RM(Nb)IC TT4 and TT8 (green circles) and reference alloys KZ5, KZ6 and JG3 (see Appendix A). AC data R^2^ = 0.9666 and HT data R^2^ = 0.6153. The parameters were calculated as described in [12,13].

The hardness of the alloys exhibited maxima in the hardness versus Δχ map, Figure 18a, at Δχ about 0.163. From the hardness versus δ or VEC maps (figures not shown) the corresponding values of these parameters, respectively are approximately 13.7 and 4.41. Figure 18b shows the hardness versus δ map for the B containing RM(Nb)ICs alloys TT4 to TT8 and the B free reference RM(Nb)ICs KZ5, KZ6, and JG3. In this figure, the fit of the data to the fourth order polynomial shows local minimum and maximum of hardness at approximately the same value of 700 HV for δ about 13.3 for the data of the B containing RM(Nb)ICs and RM(Nb)ICs/RCCAs in the AC and HT conditions.

The room temperature specific strength of the alloys of this study calculated from the hardness data (σ_y_ = (1/3) HV and density from Table 2) was in the ranges 339 to 393 and 311 to 371 MPa cm^3^ g^−1^ for the AC and HT conditions, respectively. The alloy TT6 had the highest specific strength in both conditions. The aforementioned specific strengths are higher than those of RCCAs studied to date [2] and comparable with those of RM(Nb)ICs and RM(Nb)ICs/RCCAs studied to date [1,4,21,24].

**Figure 19:**
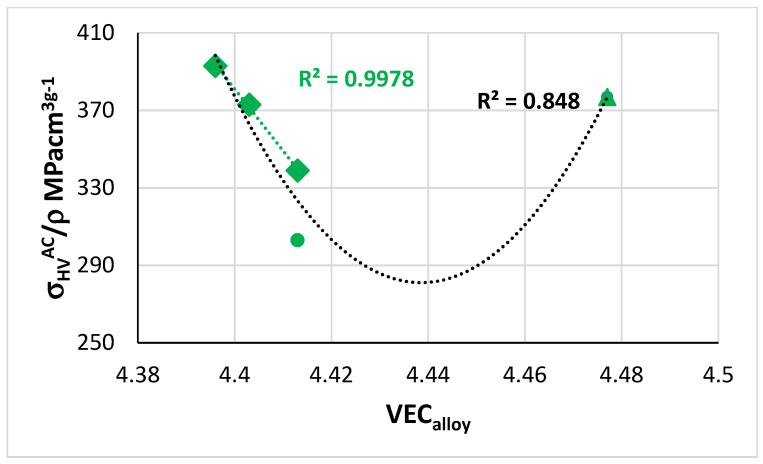
Room temperature specific strength calculated from hardness for AC RM(Nb)ICs/RCCAs TT5, TT6, TT7 (diamonds), TT4 (circle) and TT8 (triangle). Linear fit of data for TT4 to TT7 gives R^2^ = 0.848, parabolic fit of all the data R^2^ = 0.848. The parameter VEC was calculated as described in [13].

The specific strength versus VEC map of the alloys TT4 to TT8 is shown in Figure 19. All data exhibits a minimum for VEC about 4.44, whereas the data for the RM(Nb)ICs/RCCAs TT5 to TT7 has VEC around 4.4. NICE suggest that B containing RM(Nb)ICs/RCCAs with TM, RM, and simple metal and metalloid additions and with δ ≈ 13.3, Δχ ≈ 0.16 and VEC ≈ 4.4 would offer balance of creep and oxidation properties with density 6.4 < ρ < 7 g cm^−3^ and specific room temperature strength equal to or greater than 330 MPa cm^3^ g^−1^.

#### 4.3.2. Oxidation

**Figure 20:**
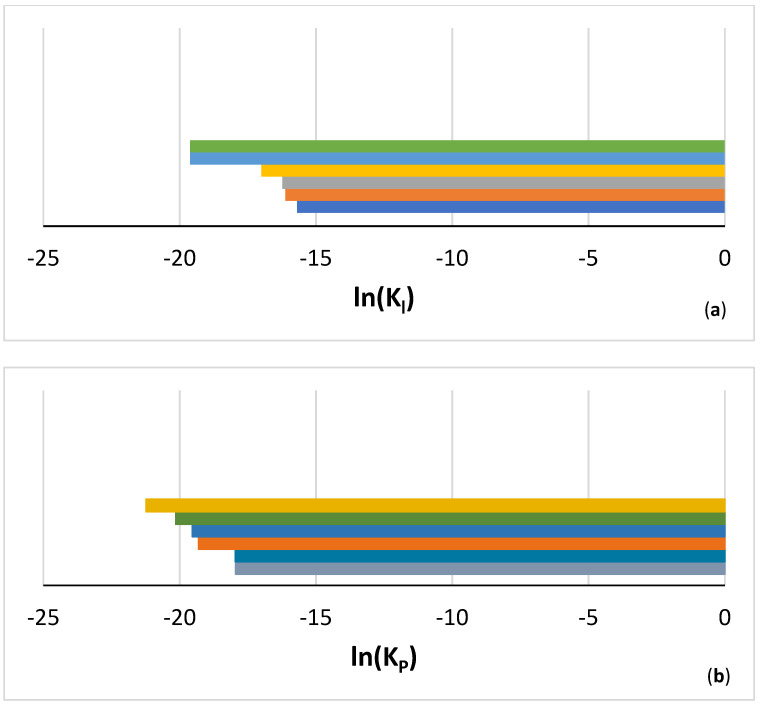
Comparison of the oxidation rate constants of the alloys in Figure 2: (**a**) ln(K_l_) at 800 °C, (**b**) ln(K_p_) at 1200 °C. Same colours are used for the alloys in (**a**,**b**). From bottom to top in (**a**) MASC, TT5, TT4, TT7, TT6, TT8 and in (**b**) TT4, MASC, TT5, TT8, TT6, TT7.

Similarly with the synergy of B with Mo in TT8 [27], the synergy of B with Hf (TT7) or Sn (TT6) suppressed pest oxidation at 800 °C and scale spallation at 1200 °C, compared with the synergy of B with Ta (TT5) that suppressed pest oxidation but not scale spallation at both temperatures (Table 7 and Figure 3). Comparison of the latter alloy with the reference alloy KZ6 shows that the addition of B reduced the mass changes at both temperatures (38 and 65 mg/cm^2^ for KZ6). Thus, the synergy of B with Mo in TT8 was more effective regarding oxidation at both temperatures, compared with the synergy of B with Ta. In TT6, the synergy of B with Sn achieved the suppression of pest oxidation, as was the case in ZX8 with the addition of Sn [29] and stopped scale spallation at 1200 °C, whereas the synergy of B with Hf in TT7, compared with the addition of Hf in JN1 [30], suppressed pesting and scale spallation at 800 °C and improved the adhesion of the scale at 1200 °C that resulted to complete suppression of scale spallation. The mass change in TT7 at 1200 °C was significantly lower than those of the alloys AC6 (Nb-24Ti-18Si-2Al-2Cr-2Hf-6B) and AC7 (Nb-24Ti-18Si-2Al-4Cr-2Hf-4B) in [9], which were 130 and 85 mg/cm^2^, respectively. At 1200 °C the lowest oxidation rate constants were exhibited by the alloys TT6 and TT7, and at 800 °C by TT6 and TT8 (Figure 20). At both temperatures, the alloying with Ta (TT5), Sn (TT6), Hf (TT7), and Mo (TT8) improved oxidation compared with the alloys MASC and TT4 (Table 7 and Figure 20).

Compared with the alloy TT5, the alloy TT8 had similar vol.% of the Nb_ss_ (Table 2, [27]) and D8_8_ silicide whereas the alloy TT6 had lower vol.% of D8_8_ and the TT7 had lower vol.% of both phases. At 800 °C, the oxidation of TT8 was better than TT7 but the reverse was the case at 1200 °C. In general, increasing the vol.% of bcc Nb_ss_ is harmful for oxidation at both temperatures [1,3,4] and for creep [3]. Hexagonal Nb_5_Si_3_ has inferior creep than tetragonal Nb_5_Si_3_ [3,14]. The data for the B containing RM(Nb)ICs and RM(Nb)ICs/RCCAs point to the need for further research to clarify the role of the D8_8_ silicide in the oxidation and creep of these alloys at low, intermediate and high temperatures.

Comparison of the alloys ZF9 [49] and TT7 shows that the synergy of Hf with Ge is significantly more effective at 800 °C (ZF9, no pest, and parabolic oxidation) compared with that of B with Hf, but not at 1200 °C (scale spallation in ZF9).

**Figure 21:**
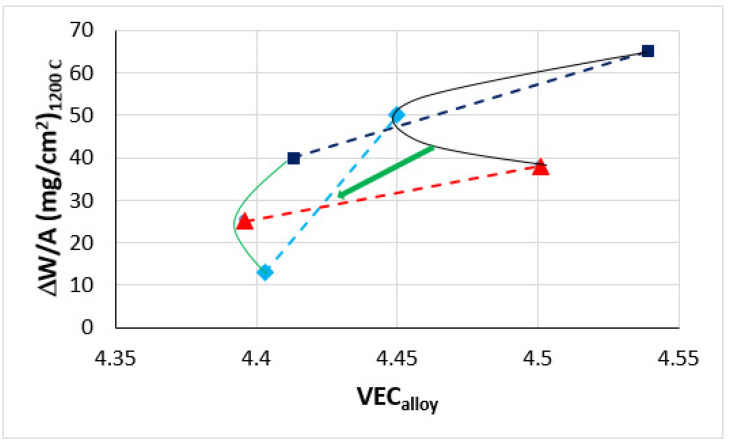
Mass change (ΔW/A) at 1200 °C versus alloy parameter VEC for the alloys KZ6 and TT5 (squares), ZX8 and TT6 (triangles), and JN1 and TT7 (diamonds). Arrow points “direction of change” for improved oxidation. The parameter VEC was calculated as described in [13].

The mass change at 1200 °C versus VEC map of the alloys TT5, TT6, TT7, KZ6, ZX8, and JN1 is shown in Figure 21. According to NICE, the oxidation of RM(Nb)ICs and RM(Nb)ICs/RCCAs improves as the value of the parameter VEC decreases. This is supported by the results of this work. The green arrow in Figure 21 indicates the “direction of change” of VEC from the “domain” of B free RM(Nb)ICs (thin black line) to that of B containing RM(Nb)ICs/RCCAs (thin green line). The map in Figure 21 is another example of how NICE links “conventional” metallic UHTMs with HEAs or RCCAs, such as, for example, the δ versus VEC and Δχ versus VEC maps in Figure 13 in [22] or maps of aforementioned parameters in Figure 12 in [20] or Figure 16 and Figure 19 in [1]. Note that the usefulness of parameter maps constructed using NICE for alloys, their phases and bond coat alloys for environmental coatings was discussed in [1,4,21,22].

#### 4.3.3. Comparison with Alloy Design Goals and Constraints

The property targets and microstructure constraints used in NICE for the design of the alloys were: (a) minimum room temperature strength of 2000 MPa; and (b) mass change (ΔW/A) of 5 and 25 mg/cm^2^ in isothermal oxidation, respectively at 800 and 1200 °C. The former target was achieved for all alloys. Indeed, the yield strength (σ_HV_^RT^, MPa) calculated from Vickers hardness was 2337, 2537, and 2537 MPa for the cast and 2148, 2386, and 2151 MPa for the heat treated TT5, TT6, and TT7 alloys, respectively. The oxidation target for 800 °C was achieved only for the alloy TT6. The target for 1200 °C was achieved only for the alloys TT6 and TT7.

The alloy design had four constraints, namely, (a) Ti/Si = 1.4; (b) stable phases bcc Nb_ss_ and the tetragonal T2 and hexagonal D8_8_ silicides; (c) the alloys to be in the area C in the Δχ versus δ master map of metallic UHTMs [1]; and (d) the macrosegregation of Si and Ti to be less than 5 and 4 at.%, respectively. All alloys had Ti/Si ratio close to 1.4 (Table 1), but only the alloy TT7 met this constraint (nominal and heat-treated composition). Metallurgists using cold hearth melting technologies to make RM alloys with TM and simple metal and metalloid element additions with different melting temperatures have to face up to the loss of low melting point elements, which makes it difficult to get the actual alloy composition matching the nominal one. In RM(Nb)ICs and RM(Nb)ICs/RCCAs, this difficulty is often demonstrated by the Ti/Si, Al/Cr, and Sn/Ge ratios differing from those in the nominal alloy composition [24,34]. The bcc Nb_ss_ and the tetragonal T2 and hexagonal D8_8_ silicides were stable phases only in the heat-treated alloys TT5 and TT7, whereas in TT6-HT the Nb_3_Sn was stable instead of the solid solution. All three alloys were indeed located in the area C in the Δχ versus δ master map of metallic UHTMs [1] as they had Δχ in the range 0.155 to 0.172 and δ in the range 12.57 to 13.86. All three alloys satisfied the MACSi and MACTi constraints (Table 3).

## 5. Conclusions

We studied the effect of the synergy of B with a metalloid element (Sn), a TM (Hf) and a RM (Ta) on the density, macrosegregation, microstructure, hardness, and oxidation of three RM(Nb)ICs/RCCAs, namely, the alloys TT5, TT6, and TT7. In actual fact, these alloys were based on the B containing RM(Nb)IC alloy TT4 to which Ta (TT5), Sn (TT6), or Hf (TT7) was added. This choice of alloys made it possible to compare the effect of the synergy of B with each of the aforementioned elements on microstructure and properties with the B free reference RM(Nb)ICs KZ6 (Ta), ZX8 (Sn), and JN1 (Hf).

The macrosegregation of B was reduced with the addition of Hf, Sn, or Ta, of Ti with the addition of Sn or Ta and of Si with the addition of Hf or Sn. All three alloys had densities less than 7 g/cm^3^, the TT5 and TT6 had the higher and lowest density, respectively, and the density of the latter was slightly higher than that of TT4. The alloy TT6 had the highest specific strength in the AC and HT conditions, which was also higher than that of RCCAs and RHEAs. The Nb_ss_ and the T2 and D8_8_ silicides were stable in the alloys TT5 and TT7, and also with TT4, whereas in the alloy TT6, the stable phases were the A15-Nb_3_Sn and the T2 and D8_8_ silicides. In all alloys, the latter silicide was Al free. The T2 had Si + B + Al ≈ 37.5 at.%, Si/B ≈ 5 and <Nb>/<Si> ≈ 1.65 compared with Si + B ≈ 40.4 at.%, Si/B ≈ 0.5, and <Nb>/<Si> ≈ 1.4 of the D8_8_. Similar with TT4, all three alloys did not pest at 800 °C, but at this temperature only the scale that was formed on TT5 spalled off. At 1200 °C, the scale of TT5 spalled off, comparably with TT4, but not the scales of TT6 and TT7. Compared with the B free alloys, and with the alloy TT8 that is also based on TT4 and with the Mo addition, the synergy of B with Ta was the least effective at 800 and 1200 °C. Considering specific strength, oxidation, and macrosegregation, the alloy TT6 offered the best balance of properties.

The macrosegregation of Si and Ti, the chemical composition of phases, the microhardness of Nb_ss_ and the hardness of the alloys, and the oxidation of the alloys at 800 and 1200 °C were all viewed from the perspective of the alloy design methodology NICE and their relationships with the parameters, VEC, δ, and Δχ, of the alloy or phase. The trends of these parameters and the location of the alloys and phases in the parameter maps were found to be in agreement with NICE.

## Figures and Tables

**Table 1:** Nominal alloy compositions and actual average compositions of the as cast (AC) and heat treated (HT) alloys.

Alloy	Condition	Element (at.%)									Ti/Si
		Nb	Ti	Si	Al	B	Cr	Hf	Sn	Ta	
TT5	nominal	37	24	18	5	5	5	-	-	6	1.33
	AC	38.6	23.7	16.4	4.6	6.3	4.9	-	-	5.5	1.45
	HT	38.6	23.5	17.3	4.4	5.5	4.9	-	-	5.8	1.36
TT6	nominal	39	24	18	4	6	5	-	4	-	1.33
	AC	39.3	23.7	17.5	3.4	6.7	5.2	-	4.2	-	1.35
	HT	39.3	23	18.4	3.6	6.3	5.3	-	4.1	-	1.25
TT7	nominal	38	24	17	5	6	5	5	-	-	1.41
	AC	40.6	23.3	17	4.5	5.2	4.7	4.7	-	-	1.37
	HT	39	24.1	17.1	4.4	6.1	4.6	4.7	-	-	1.41

**Table 2:** Density (ρ) of alloys, vol.% Nb_ss_, hardness of alloys, and microhardness of Nb_ss_ and T2 silicide. The hardness data gives the average value, the standard deviation, and the minimum and maximum measured values.

Alloy	Hardness HV_alloy_		ρ (g/cm^3^)		Vol.% Nb_ss_		Microhardness HV_phase_			
							Nb_ss_		T2	
	AC	HT	AC	HT	AC	HT	AC	HT	AC	HT
TT5	715 ± 53 645 − 799	657 ± 31 600 − 693	6.9	6.9	32	38	576 ± 28 538 − 631	503 ± 14 479 − 521	1480 ± 57 1369 − 1572	1484 ± 62 1369 − 1572
TT6	776 ± 43 712 − 842	730 ± 33 684 − 782	6.46	6.43	29 *	43 *	758 ± 28 720 − 796 **^◊^	678 ± 28 631–721 **^◊^	1378 ± 63 1265 − 1468	1214 ± 42 1143 − 1265
TT7	776 ± 55 696 − 866	658 ± 32 612 − 708	6.8	6.83	10	37	539 ± 22 512 − 577	476 ± 21 437 − 504	1340 ± 33 1296 − 1405	1200 ± 55 1116 − 1299

* This is the vol.% of Nb_3_Sn, ** this is the microhardness of Nb_3_Sn, ^◊^ it was not possible to measure the microhardness of the Nb_ss_ in TT6.

**Table 3:** Macrosegregation (at.%) of solutes MACX (X = Al, B, Cr, Si, Sn, Ti) in the cast alloys, where MACX = C_max_^X^ − C_min_^X^ [32].

Alloy	MACX (at.%)					
	Al	B	Cr	Si	Ti	Sn
TT5	1.3	2.4	1.3	4.8	1.6	-
TT6	1.2	2.5	2.3	3.9	3	1.1
TT7	-	2.8	2.2	1.7	2.4	-

**Table 4:** Phases in the as cast (AC) and heat treated (HT) alloys according to the XRD and EPMA data.

Alloy and Condition	Nb_ss_	T2	Cr and Ti Rich Phase	C14 Laves	Nb_ss_ + T2 Eutectic **	Nb_3_Sn	Nb_3_Si	D8_8_	Nb_5_Si_3_	Nb_ss_ + Nb_5_Si_3_ Eutectic
TT5-AC	X *	X *	X	-	X	-	X	X	-	-
TT5-HT	X	X *	-	-	-	-	-	X	-	-
TT6-AC	X ^◊^	X *	-	-	X	X	X	X	-	-
TT6-HT	-	X *	-	-	-	X	-	X	-	-
TT7-AC	X *	X *	-	-	-	-	X	X	-	-
TT7-HT	X	X	-	-	-	-	-	X	-	-
KZ6-AC	X *	-	-	-	-	-	-	-	X *, β	X
KZ6-HT	X	-	-	-	-	-	-	-	X *, β & α	-
ZX8-AC	-	-	-	X	-	X	-	-	X *, β	-
ZX8-HT	X	-	-	X	-	X	-	-	X *, α	-
JN1-AC	X *	-	-	-	-	-	-	-	X *, β	X
JN1-HT	X	-	-	-	-	-	-	-	X *, β	-

* Indicates the presence also of Ti rich phase, ^◊^ indicates only Ti rich Nb_ss_ (see Table 5), ** present at very small vol.%.

**Table 5:** EPMA data (at.%) for the phases in the AC and HT alloys TT5, TT6, and TT7.

Alloy	Phase	Nb	Ti	Si	B	Cr	Al	Hf	Sn	Ta
TT5-AC	Nb_ss_	41.5 − 46.1 43.5 ± 0.8	29.1 − 32.3 31.1 ± 0.6	1.2 − 1.7 1.5 ± 0.2	0 − 1.6 0.7 ± 0.4	8.1 − 10.4 7.1 ± 0.4	5.2 − 7.7 6.7 ± 0.4	-	-	6.5 − 7.7 7.0 ± 0.4
	Ti rich Nb_ss_	27.9 − 34.1 31.4 ± 1.5	38.1 − 43.6 40.2 ± 1.5	1.3 − 1.9 1.4 ± 0.3	-	14.5 − 17.3 15.9 ± 0.4	5.8 − 7.9 7.1 ± 0.6	-	-	3 − 5.3 3.7 ± 0.3
	T_2_	34.4 − 35.7 35.2 ± 0.3	19.1 − 22.8 20.9 ± 0.9	29.6 − 31.9 29.2 ± 1.3	3.1 − 5.7 6.2 ± 1.2	0.4 − 0.9 0.7 ± 0.2	2.2 − 3.9 2.4 ± 0.6	-	-	4.9 − 5.8 5.4 ± 0.4
	Ti rich T_2_	22.9 − 28.1 25.4 ± 2.4	29.5 − 36.5 32.8 ± 3.8	26.5 − 29 27.7 ± 0.9	3.8 − 6 5.4 ± 0.3	1.6 − 2.6 2.2 ± 0.5	3.5 − 4.3 3.9 ± 0.3	-	-	2.0 − 3.2 2.6 ± 0.6
	D8_8_	38.1 − 39.5 38 ± 0.9	11.2 − 12.5 12 ± 0.2	14.6 − 16.1 14.2 ± 0.6	22.1 − 26.7 25.8 ± 0.5	0.8 − 1.4 1.1 ± 0.1	-	-	-	8.5 − 9.2 8.9 ± 0.3
TT6-AC	Nb_3_Sn	41.1 − 46.3 43.7 ± 1.7	28.2 − 31.1 29.9 ± 1.9	3.0 − 3.3 3.1 ± 0.1	0.4 − 4.2 2.4 ± 1.4	3.5 − 4.8 4.3 ± 0.4	3.7 − 5.5 4.6 ± 0.3	-	11.0 − 12.4 11.8 ± 0.7	-
	Ti rich Nb_ss_	26.2 − 29.9 28.0 ± 1.0	45.5 − 49.3 47.0 ± 0.7	0.5 − 1.4 0.9 ± 0.5	-	10.2 − 13.7 12.1 ± 0.4	7.3 − 8.7 8.1 ± 0.3	-	3.5 − 4.7 4.1 ±0.1	-
	T_2_	37.9 − 41 39.9 ± 0.7	19.6 − 20.8 20 ± 0.6	28.5 − 30.8 29.6 ± 1.4	5.7 − 9.4 6.9 ± 1.7	0.6 − 9.4 0.9 ± 0.1	0.6 − 1.2 2.1 ± 0.3	-	1.5 − 2.9 0.6 ± 0.1	-
	Ti-rich T_2_	30.7 − 34.6 33.0 ± 0.9	24.8 − 29.4 27.1 ± 0.6	26.4 − 28.4 27.4 ± 0.4	4.9 − 6.8 5.8 ± 0.4	1.0 − 1.8 1.4 ± 0.3	3.7 − 4.8 4.3 ± 0.3	-	0.7 − 1.1 0.8 ± 0.1	-
	D8_8_	42.8 − 44.8 44.1 ± 2.3	11.5 − 12.9 12.1 ± 0.5	13.1 − 15.2 14.2 ± 1.9	27.2 − 28.5 28.2 ± 4.1	0.9 − 2.1 1.2 ± 0.2	-	-	-	-
TT7-AC	Nb_ss_	44.3 − 50.3 47.4 ± 3.3	30.0 − 32.2 31.2 ± 1.1	0.9 − 1.2 1.0 ± 0.3	0 − 7.3 2.4 ± 1.8	7.1 − 10.2 8.9 ± 1.2	4.8 − 6.8 5.8 ± 1.3	3.1 − 3.3 3.2 ± 0.3	-	-
	Ti rich Nb_ss_	34.8 − 40.8 38.2 ± 2.3	32.7 − 40.4 36.7 ± 3.3	0.4 − 1.6 0.9 ± 0.4	0 − 4.5 3.0 ± 2.5	10.4 − 13.4 11.9 ± 1.3	4.6 − 6.7 5.6 ± 1.3	3.4 − 3.7 3.5 ± 0.2	-	-
	T_2_	32.0 − 37.3 34.7 ± 2.3	16.7 − 20.6 19.1 ± 1.1	27.8 − 31.9 29.8 ± 1.5	1.6 − 11.3 6.0 ± 3.2	0.5 − 1.4 0.9 ± 0.1	2.0 − 4.6 3.5 ± 2.0	6.5 − 7.5 6.9 ± 0.6	-	-
	Ti rich T_2_	31.5 − 36 27.6 ± 3	24.8 − 28.4 26.6 ± 0.8	26.9 − 29.7 28.6 ± 0.5	1.5 − 7.9 4.7 ± 2.9	0.9 − 1.7 1.3 ± 0.3	3.7 − 4.8 4.2 ± 0.9	9.1 − 10.5 9.7 ± 0.5	-	-
TT5-HT	Nb_ss_	38.8 − 44.0 41.6 ± 2.4	27.6 − 34.9 31.1 ± 2.7	0.4 − 0.8 0.6 ± 0.2	0 − 7.4 4.1 ± 2.8	9.7 − 12.0 10.7 ± 0.5	5.7 − 6.7 6.4 ± 1.0	-	-	6.1 − 7.0 6.8 ± 0.4
	T_2_	33.8 − 38 36.4 ± 1.4	18.2 − 22.9 20.7 ± 1.9	25 − 32.5 28 ± 2.9	1.6 − 13.7 6.7 ± 4.5	0.4 − 1.1 0.6 ± 0.2	1.7 − 2.4 2.3 ± 0.3	-	-	3.6 − 6.1 5.3 ± 1.2
	Ti rich T_2_	29.7 − 33.5 31.1 ± 1.8	25.3 − 26.5 25.7 ± 0.6	28.1 − 30.9 29 ± 1.3	2.2 − 8.3 6 ± 2.9	0.5 − 1.7 1.3 ± 0.2	2.6 − 3.6 3 ± 0.2	-	-	3.7 − 4.0 3.9 ± 0.1
	D8_8_	30.4 − 37.0 38.5 ± 3.2	9.4 − 14.8 11.8 ± 2.0	10.4 − 15.9 13.9 ± 0.4	23 − 29.2 26.1 ± 2.2	0.5 − 1.6 1 ± 0.3	-	-	-	7.4 − 9.9 8.7 ± 0.4
TT6-HT	Nb_3_Sn	36.5 − 47.3 42.0 ± 3.9	28.3 − 33.3 31.1 ± 2.3	2.6 − 3.3 3.1 ± 0.3	0 − 6.4 3.6 ± 2.2	3.7 − 4.9 4.4 ± 0.5	5.1 − 6.4 5.7 ± 0.5	-	10.8 − 12.2 11.4 ± 0.5	-
	T_2_	41.4 − 44 42.5 ± 1.3	19.3 − 20.4 19.8 ± 0.6	27.2 − 31.8 28 ± 1.9	6.4 − 8.8 7.1 ± 1.2	0.3 − 0.7 0.5 ± 0.2	0.9 − 2.4 1.5 ± 0.5	-	0.4 − 0.7 0.6 ± 0.1	-
	Ti rich T_2_	31.5 − 39.8 36 ± 3.2	22.5 − 23.1 22.9 ± 0.4	24.5 − 30.9 28 ± 2.2	2.1 − 9 5.2 ± 2.5	0.6 − 1.3 1 ± 0.1	2.8 − 4.6 3.9 ± 0.9	-	0.8 − 1.2 1 ± 0.1	-
	D8_8_	40.1 − 51 45.9 ± 3.2	11.5 − 13.1 12.3 ± 0.7	10.8 − 13.1 12.1 ± 1.1	27.5 − 37.6 28 ± 4.7	0.9 − 2.4 1.7 ± 0.6	-	-	-	-
TT7-HT	Nb_ss_	51.1 − 51.9 51.6 ± 0.4	26.7 − 27.4 27.0 ± 0.4	-	3.0 − 3.8 3.4 ± 0.2	8.3 − 8.7 8.5 ± 0.2	7.1 − 7.7 7.4 ± 0.3	2.1 − 2.2 2.2 ± 0.1	-	-
	T_2_	28.0 − 30.5 29.2 ± 1	19.0 − 24.0 21.3 ± 2.5	26.9 − 29.0 27.8 ± 0.9	3.8 − 10.1 6.4 ± 3.4	0.3 − 1.0 0.6 ± 0.4	2.0 − 3.8 3.3 ± 0.9	7.8 − 8.3 8.0 ± 0.2	-	-
	Ti rich T_2_	27.3 − 32.3 29.9 ± 1.8	23.3 − 27.7 24.8 ± 1.7	27.2 − 30.2 29.5 ± 1.4	1.1 − 7.9 4.2 ± 3	0.5 − 1.0 0.8 ± 0.2	1.9 − 3.5 2.9 ± 0.7	8.2 − 9.1 8.7 ± 0.4	-	-
	D8_8_	47.6 − 49.4 45.4 ± 0.9	14.0 − 14.5 11.3 ± 0.3	13.5 − 13.8 12.7 ± 0.2	23.2 − 29.9 27.3 ± 1.8	1.4 − 1.6 1.5 ± 0.1	-	1.6 − 2 1.8 ± 0.1	-	-

**Table 6:** <Nb>/<Si> and Si/B ratios and Si + B, Si + B + Al and Si + Al + B + Sn sums in T2 and D8_8_ silicides in the AC and HT alloys, where <Nb> = Nb + TM + RM, <Si> = Al + B + Si + Sn, TM = Cr, Hf, Ti, and RM = Ta.

Alloy and Condition	Phase	Parameter				
		Si + B (at.%)	Si/B	Si + B + Al (at.%)	Si + Al + B + Sn (at.%)	<Nb/<Si>
TT5-AC	T2	35.4	4.7	37.8		1.65
	Ti rich T2	33.1	5.1	37		1.7
	D8_8_	40	0.55			1.5
TT5-HT	T2	34.7	4.2	37		1.7
	Ti rich T2 *	35	4.8	38		1.63
	D8_8_	40	0.53			1.5
TT6-AC	T2	36.5	4.3	38.6	39.2	1.55
	Ti rich T2	33.2	4.7	37.5	38.3	1.61
	D8_8_	42.4	0.5			1.36
TT6-HT	T2	35.1	3.9	36.6	37.2	1.69
	Ti rich T2	33.2	5.4	37.1	38.1	1.62
	D8_8_	40.1	0.43			1.49
TT7-AC	T2	35.8	5	39.3		1.54
	Ti rich T2	33.3	6.1	37.5		1.67
	D8_8_ *	40.2, 40.4	0.49, 0.46			1.49, 1.47
TT7-HT	T2	34.2	4.3	37.5		1.67
	Ti rich T2	33.7	7	36.6		1.73
	D8_8_	40	0.47			1.5
Average	T2	34.5	4.96	37.5	38.2	1.65
	D8_8_	40.4	0.49			1.47

* See text.

**Table 7:** Oxidation rate constants and mass changes in the alloys MASC, TT4 to TT8 after isothermal oxidation at 800 and 1200 °C. Data for MASC, TT4, and TT8 from [27]. The oxidation data are for 100 h unless stated otherwise.

Alloy	Oxidation Rate Constant		Mass Change ΔW/A (mg/cm^2^)		Pest Oxidation	Scale Spallation	
	800 °C k_l_ (g/cm^2^ s)	1200 °C k_p_ (g^2^/cm^4^ s)	800 °C	1200 °C	800 °C	800 °C	1200 °C
MASC	1.5 × 10^−7^	1.6 × 10^−8^	36 (≤65 h)	80	MC *	-	Yes
TT4	8.9 × 10^−8^	3.4 × 10^−9^	27.9	34	No	Yes	Yes
TT5	1 × 10^−7^	4.2 × 10^−9^	33.8	34 ^++^	No	Yes	Yes
TT6	3 × 10^−9^	1.8 × 10^−9^	1.4	23.5 ^+^	No	No	No
TT7	4.2 × 10^−8^	6 × 10^−10^	18.9	11.3 **	No	No	No
TT8	3 × 10^−9^	3.3 × 10^−9^	1.4	30	No	No	No

* MC = Maltese cross, ** after 68 h, ^+^ after 90 h, ^++^ after 73 h.

**Table 8:** Comparison of the macrosegregation (MACX) of X = B, Si, Ti in the B containing RM(Nb)ICs/RCCAs of this work, the RM(Nb)ICs TT4 and TT8 [27] and the RM(Nb)ICs KZ5 [34], KZ6, JG3 [35], JN1, and ZX8 (see Appendix A for nominal alloy compositions).

Alloy	MACSi (at.%)	Alloy	MACB (at.%)	Alloy	MACTi (at.%)
ZX8	10	TT8	4.9	ZX8	9.7
TT5	4.8	TT4	2.9	KZ6	3.4
TT4	4.7	TT7	2.8	TT4	3.3
KZ6	4.5	TT6	2.5	TT6	3.0
JN1	4.0	TT5	2.4	TT8	2.8
TT6	3.9			TT7	2.4
JG3	2.7			JN1	1.9
TT7	1.7			TT5	1.6
TT8	1.3			JG3	1.5
KZ5	1.3			KZ5	1.4

**Table 9:** Comparison of the Si + Sn, Si + Sn + Al and Si + Sn + Al + B concentrations and the Si/Sn, (Si + Al)/Sn, and (Si + Al + B)/Sn ratios in A15-Nb_3_Sn in AC and HT RM(Nb)ICs alloys ZX8 [29] and NV9 [40] and the RM(Nb)IC/RCCA alloy TT6. Numbers rounded up to first decimal point.

Alloy and Condition	Si + Sn (at.%)	Si + Sn + Al (at.%)	Si + Sn + Al + B (at.%)	Si/Sn	(Si + Al)/Sn	(Si + Al + B)/Sn
TT6-AC	-	19.5	21.9	-		0.9
TT6-HT	-	20.2	23.8	-		1.1
ZX8-AC	-	20.1	-	-	1.1	-
ZX8-HT	-	19.3	-	-	1.2	-
NV9-AC	17.8	-	-	1		-
NV9-HT *	17.2	-	-	0.8		-

* 1500 C/100 h.

## Data Availability

All the data for this paper is given in the paper and its Supplementary Materials, other data cannot be made available to the public.

## Supplementary Materials

The following are available online at https://www.mdpi.com/1996-1944/14/24/7615/s1, Figure S1: X ray diffractograms of the AC and HT alloys (a) and (b) TT5, (c) and (d) TT6 and (e) and (f) TT7.

## Appendix A

**Table A1:** The reference alloys used in this work.

Alloy	Nominal Composition (at.%)	Ref.
JG3	Nb-24Ti-18Si-5Al-5Cr-2Mo	[35]
JN1	Nb-24Ti-18Si-5Al-5Cr-5Hf	[30]
KZ5	Nb-24Ti-18Si-5Al-5Cr	[34]
KZ6	Nb-24Ti-18Si-5Al-5Cr-6Ta	[28]
NV6	Nb-24Ti-18Si-5Sn	[40]
NV9	Nb-18Si-5Sn	[40]
TT1	Nb-24Ti-18Si-8B	[26]
TT2	Nb-24Ti-18Si-5Cr-7B	[27]
TT3	Nb-24Ti-16Si-5Al-7B	[27]
TT4	Nb-24Ti-18Si-5Al-5Cr-8B	[27]
TT8	Nb-24Ti-17Si-3.5Al-5Cr-6B-2Mo	[27]
YG1	Nb-18Si-5Cr-5Hf	[44]
YG2	Nb-18Si-5Al-5Hf	[44]
YG3	Nb-24Ti-18Si-5Hf	[44]
ZF9	Nb-24Ti-18Si-5Al-5Cr-5Ge-5Hf	[49]
ZX7	Nb-24Ti-18Si-5Al-5Cr-2Sn	[41]
ZX8	Nb-24Ti-18Si-5Al-5Cr-5Sn	[29]
