# Supplementary figures and images for: On the Microstructure and Properties of Nb-Ti-Cr-Al-B-Si-X (X = Hf, Sn, Ta) Refractory Complex Concentrated Alloys
Source: Materials (Basel). 2021 Dec 10;14(24):7615. doi: 10.3390/ma14247615 (PMC8715753; doi:10.3390/ma14247615)

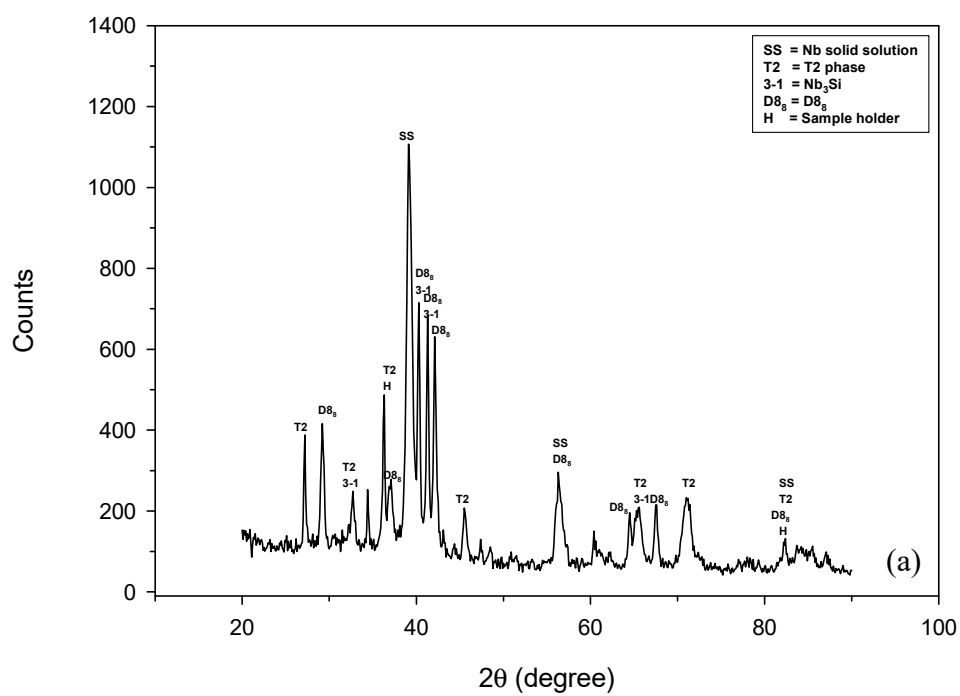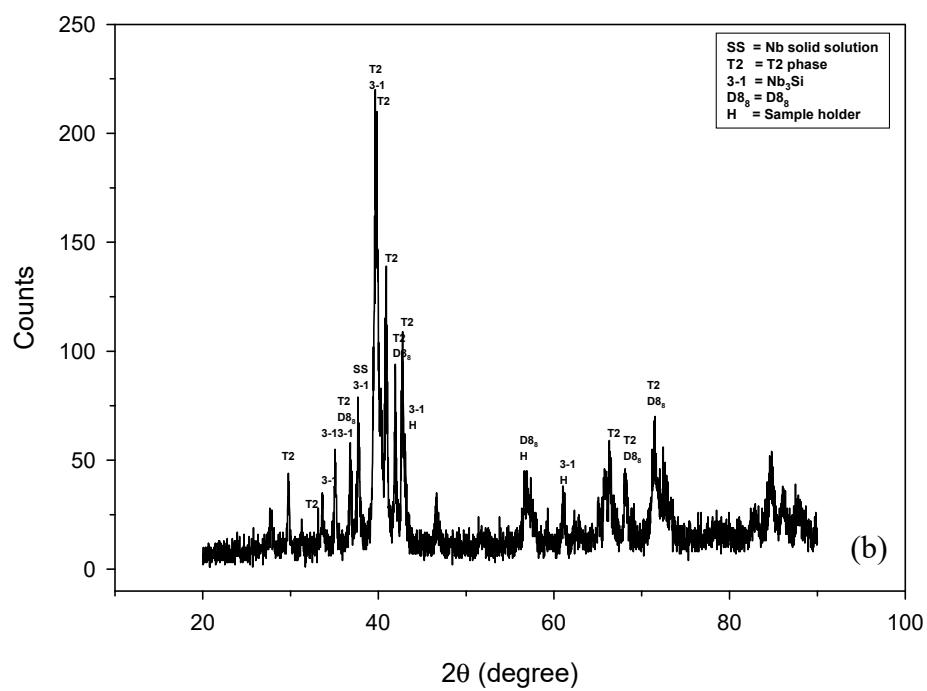

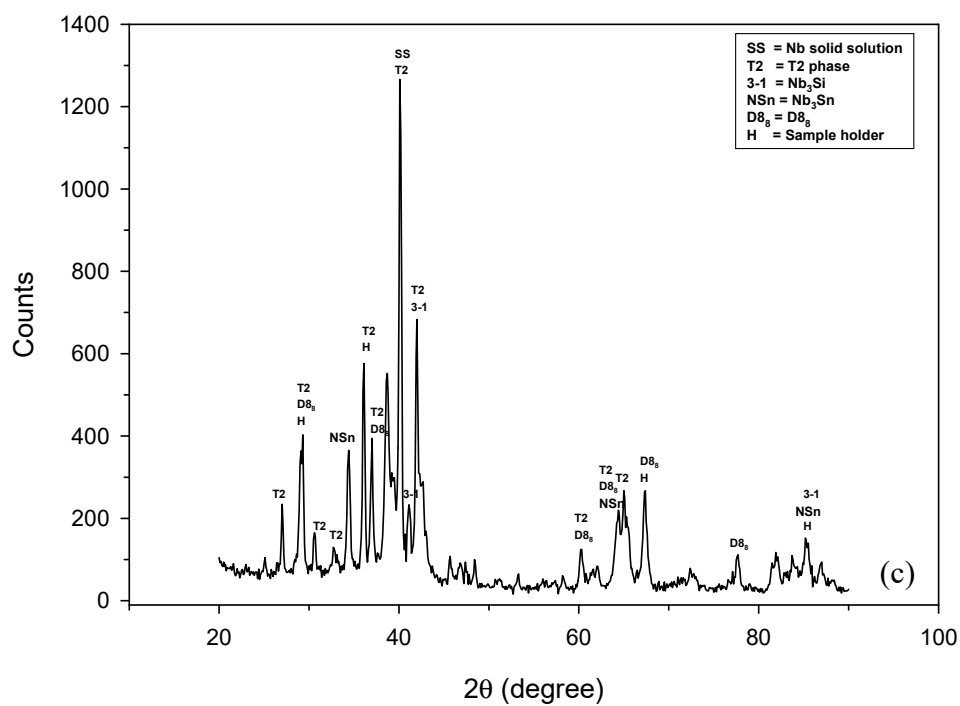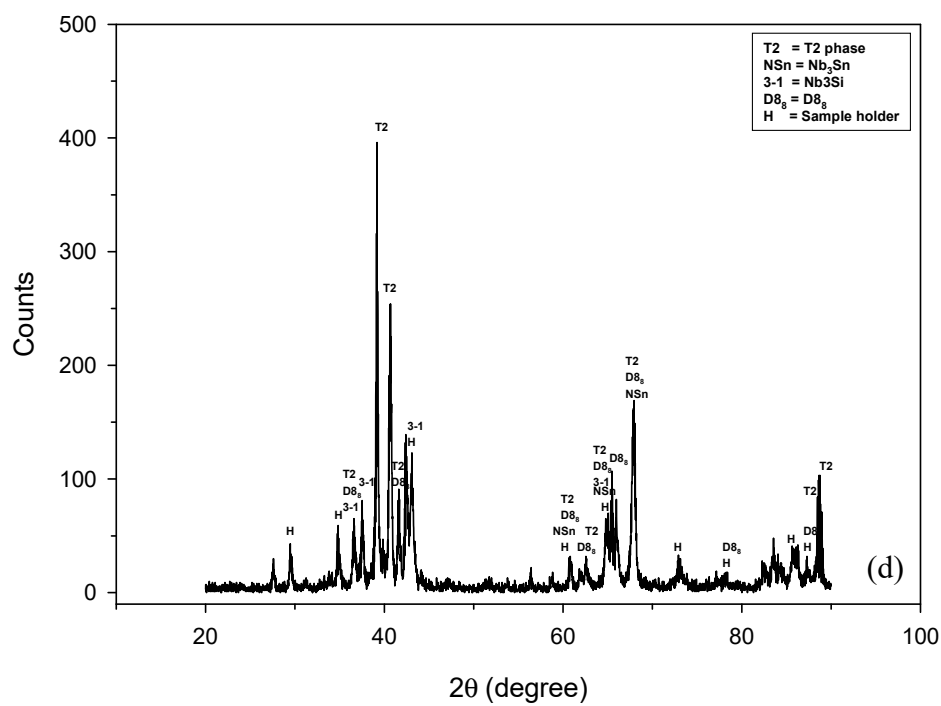

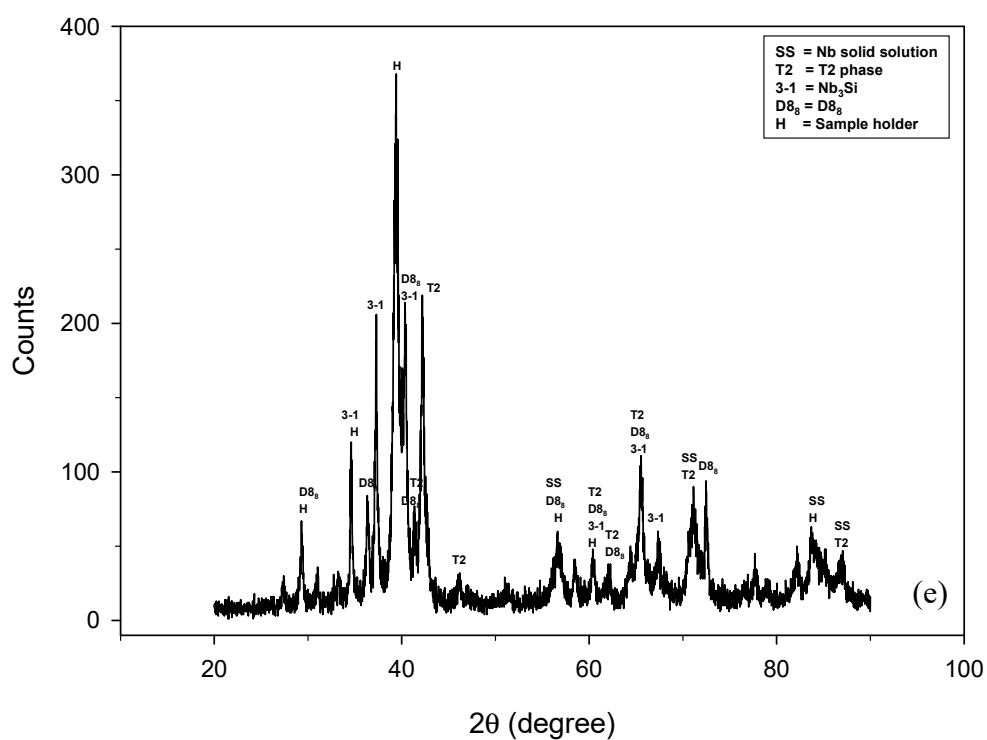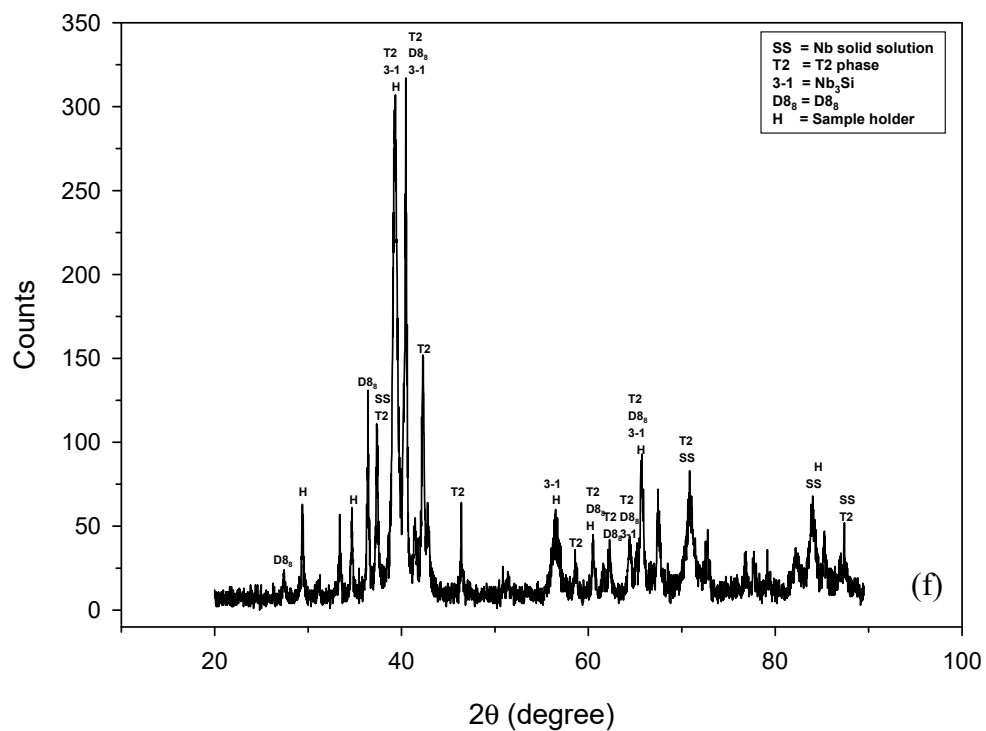

**Figure S1:** X ray diffractograms of the AC and HT alloys (a) and (b) TT5, (c) and (d) TT6 and (e) and (f) TT7.

Supplement: Supplementary file 1 [file materials-14-07615-s001.zip › materials-1455997-supplementary.pdf]
